# Diabetes Prevention and Weight Loss with a Fully Automated Behavioral Intervention by Email, Web, and Mobile Phone: A Randomized Controlled Trial Among Persons with Prediabetes

**DOI:** 10.2196/jmir.4897

**Published:** 2015-10-23

**Authors:** Gladys Block, Kristen MJ Azar, Robert J Romanelli, Torin J Block, Donald Hopkins, Heather A Carpenter, Marina S Dolginsky, Mark L Hudes, Latha P Palaniappan, Clifford H Block

**Affiliations:** ^1^ Turnaround Health a division of NutritionQuest Berkeley, CA United States; ^2^ Division of Community Health and Human Development School of Public Health University of California, Berkeley Berkeley, CA United States; ^3^ Palo Alto Medical Foundation Research Institute Palo Alto, CA United States; ^4^ Center for Weight and Health University of California, Berkeley Berkeley, CA United States

**Keywords:** type 2 diabetes, prevention, intervention studies, prediabetes, behavior change, obesity, physical activity, nutrition, Internet, smartphone, weight loss

## Abstract

**Background:**

One-third of US adults, 86 million people, have prediabetes. Two-thirds of adults are overweight or obese and at risk for diabetes. Effective and affordable interventions are needed that can reach these 86 million, and others at high risk, to reduce their progression to diagnosed diabetes.

**Objective:**

The aim was to evaluate the effectiveness of a fully automated algorithm-driven behavioral intervention for diabetes prevention, Alive-PD, delivered via the Web, Internet, mobile phone, and automated phone calls.

**Methods:**

Alive-PD provided tailored behavioral support for improvements in physical activity, eating habits, and factors such as weight loss, stress, and sleep. Weekly emails suggested small-step goals and linked to an individual Web page with tools for tracking, coaching, social support through virtual teams, competition, and health information. A mobile phone app and automated phone calls provided further support. The trial randomly assigned 339 persons to the Alive-PD intervention (n=163) or a 6-month wait-list usual-care control group (n=176). Participants were eligible if either fasting glucose or glycated hemoglobin A1c (HbA1c) was in the prediabetic range. Primary outcome measures were changes in fasting glucose and HbA1c at 6 months. Secondary outcome measures included clinic-measured changes in body weight, body mass index (BMI), waist circumference, triglyceride/high-density lipoprotein cholesterol (TG/HDL) ratio, and Framingham diabetes risk score. Analysis was by intention-to-treat.

**Results:**

Participants’ mean age was 55 (SD 8.9) years, mean BMI was 31.2 (SD 4.4) kg/m^2^, and 68.7% (233/339) were male. Mean fasting glucose was in the prediabetic range (mean 109.9, SD 8.4 mg/dL), whereas the mean HbA1c was 5.6% (SD 0.3), in the normal range. In intention-to-treat analyses, Alive-PD participants achieved significantly greater reductions than controls in fasting glucose (mean –7.36 mg/dL, 95% CI –7.85 to –6.87 vs mean –2.19, 95% CI –2.64 to –1.73, *P*<.001), HbA1c (mean –0.26%, 95% CI –0.27 to –0.24 vs mean –0.18%, 95% CI –0.19 to –0.16, *P*<.001), and body weight (mean –3.26 kg, 95% CI –3.26 to –3.25 vs mean –1.26 kg, 95% CI –1.27 to –1.26, *P*<.001). Reductions in BMI, waist circumference, and TG/HDL were also significantly greater in Alive-PD participants than in the control group. At 6 months, the Alive-PD group reduced their Framingham 8-year diabetes risk from 16% to 11%, significantly more than the control group (*P*<.001). Participation and retention was good; intervention participants interacted with the program a median of 17 (IQR 14) of 24 weeks and 71.1% (116/163) were still interacting with the program in month 6.

**Conclusions:**

Alive-PD improved glycemic control, body weight, BMI, waist circumference, TG/HDL ratio, and diabetes risk. As a fully automated system, the program has high potential for scalability and could potentially reach many of the 86 million US adults who have prediabetes as well as other at-risk groups.

**Trial Registration:**

Clinicaltrials.gov NCT01479062; https://clinicaltrials.gov/ct2/show/NCT01479062 (Archived by WebCite at http://www.webcitation.org/6bt4V20NR)

## Introduction

In the United States, 86 million adults have prediabetes [1], a condition characterized by elevated blood glucose that is not yet high enough to be diagnosed as diabetes. Chronic elevated blood glucose levels tend to increase over time and it is estimated that as many as 70% of those with prediabetes will eventually progress to type 2 diabetes [2]. The economic burden of the combined costs of diabetes and prediabetes exceeded US $322 billion in 2012 and accounted for 1 in 10 US health care dollars. In an editorial, Cefalu et al [3] noted that “increased prevalence, not increased cost per patient, is the driving force behind the increased economic burden of diabetes” [4]. Unless changes are made to prevent progression to type 2 diabetes, costs relating to diabetes management and care will continue to rise at alarming rates. It is critical to develop affordable and effective interventions that can reach more of the 86 million people with prediabetes with programs to improve glycemic control.

Lifestyle modification has been shown to reduce risk of progression to diabetes by as much as 40% to 70% [2]. The Diabetes Prevention Program (DPP) achieved a 58% reduction in the incidence of diabetes through increased physical activity, dietary changes, and weight loss [5]. The DPP involved intensive counseling and multiple in-person and group meetings in a research context. Since then, numerous translations of the DPP have been developed that attempt to provide approaches that can be widely applied.

Some adaptations of the DPP for real-world settings deliver the interventions through group meetings and in-person contact, such as those delivered in communities and YMCAs [6-8]. Ali et al [9] found a mean 4.3% body weight loss in programs delivered by medical professionals and 3.2% weight loss for those delivered by community members. Although in-person and group-based interventions are important and effective resources, barriers to widespread adoption of such programs include lack of professional staff, institutional resources, substantial costs, and the requirement that participants attend a series of in-person meetings, which together substantially limit their scalability and reach [10,11].

A number of interventions have combined some form of human coaching with the use of technology, at least by phone or email, thus enabling them to achieve wider reach. In a meta-analysis of programs modeled on the DPP, Ali et al [9] found that among electronic media-assisted programs, there was a statistically significant mean weight loss of 4% body weight. A review by Levine et al [12] of technology-assisted weight loss interventions in primary care found a mean weight loss in the intervention group of -2.7 kg among technology-assisted weight loss interventions that included some human coaching. Human feedback and coaching can provide value and effectiveness—and indeed is needed by some participants. However, it does result in higher costs that once again limit the number of persons with prediabetes that can be reached.

Fully automated behavioral intervention systems, those without any human coaching or facilitation, may hold substantial promise in overcoming barriers to widespread reach and adoption in a resource-limited health care environment if they can be shown to be effective. Several such programs have been found to be effective for weight loss [13,14], but there is very little information on the impact of such programs on glycemic markers critical for diabetes prevention. The Alive-PD intervention (Turnaround Health, a Division of NutritionQuest, Berkeley, CA, USA) provides such a fully automated, tailored, online behavior change program. Alive-PD is focused on reducing diabetes risk by reducing the biomarkers that constitute the criteria for diabetes, glycated hemoglobin A_1c_ (HbA_1c_) and fasting glucose, in persons at risk of developing diabetes. The purpose of this analysis is to examine the effects of this automated program on those glycemic biomarkers and weight loss in a randomized controlled trial.

## Methods

The Alive-PD study was a randomized, wait-list controlled (usual care) trial among patients with clinical evidence of prediabetes. The primary outcome measures were changes in HbA_1c_ and fasting glucose at 6-month follow-up from baseline. Secondary outcomes were changes in body weight, body mass index (BMI), waist circumference, triglyceride (TG) to high-density lipoprotein cholesterol (HDL-C) ratio (a proxy measure for insulin resistance [15]), and metabolic syndrome. Metabolic syndrome was defined as 3 or more of 5 components (ie, abdominal obesity, elevated blood pressure, elevated TG, low HDL, and dysglycemia) specified by the American Heart Association and the National Heart, Lung, and Blood Institute [16]. The Framingham 8-year diabetes risk score was calculated [17]. Sample size was determined by using the estimated standard deviation of change in HbA_1c_ from an intervention study on patients with diabetes [18]. With a standard deviation of 1.4 and alpha of .05, we estimated that a final sample of 268 participants would provide 80% power to detect a minimum detectable difference in change in HbA_1c_ of 0.48%. The goal for enrollment was 314 persons to achieve a sample size of 268 after 15% estimated attrition. The trial design and methods are described in detail elsewhere [19] and are summarized here (see Multimedia Appendix 1 for CONSORT flow diagram).

### Participant Recruitment and Eligibility Criteria

Potential participants whose recent fasting glucose and/or HbA_1c_ were within the prediabetes range were initially identified through an electronic health record query of patients in an ambulatory care health care delivery system, the Palo Alto Medical Foundation (PAMF). The PAMF is a community-based multispecialty group practice in Northern California. Patients meeting these criteria were recruited via letter and underwent telephone screening for eligibility. Those meeting preliminary criteria were invited to attend a clinic visit to confirm eligibility, which also provided the baseline data for those confirmed eligible. At that visit, fasting glucose and lipids were measured by point-of-care whole blood testing using the Alere Cholestech LDX Analyzer. Similarly, HbA_1c_ was measured using the Siemens DCA Vantage Analyzer. Biometric measurements, including height, body weight, waist circumference, and blood pressure were also obtained. BMI (kg/m^2^) was calculated from height and body weight.

Individuals were eligible if they were aged between 30 and 69 years with a BMI of at least 27 kg/m^2^ (BMI >25 kg/m^2^ for Asian participants) [20], spoke English, were not taking diabetes medications, had access to email and Internet, and had either fasting glucose or HbA_1c_ in the prediabetes range (glucose: 5.55-6.94 mmol/L or 100-125 mg/dL; HbA_1c_: 39-46 mmol/mol or 5.7%-6.4%). If one measure reached the diabetic range and the other was prediabetic, the patient’s primary care physician decided whether the patient had prediabetes and was eligible for the study. Additional exclusion criteria are described elsewhere [19]. The study was approved by independent Institutional Review Boards of Turnaround Health and PAMF.

After participants provided signed informed consent, they were given brief (5-10 minutes) instruction that they were at risk for developing diabetes and that increased physical activity and changes in their dietary behaviors could help prevent progression to diabetes. PAMF research staff assisted participants in signing into an account for the Alive-PD Web-based program, where participants provided their email address and password to the system. All subsequent communications with participants came from the electronic Alive-PD program and interactions with the Alive-PD program took place outside of the clinic.

### Randomization

After leaving the study site, enrolled participants completed a brief questionnaire online, which provided information required for randomization. Randomization was conducted automatically, by computer algorithm, with stratification by sex, race/ethnicity (non-Hispanic white/other), and BMI (<35 kg/m^2^/≥35 kg/m^2^), to achieve balance on those factors. Participants were randomized to start the intervention immediately (intervention group) or after 6 months’ delay (control group/wait-listed usual care group). Participants were notified of treatment group assignment by automated email from the Alive-PD system. The research and clinical staff at PAMF was masked to group assignment. Participants in the control group received no further contact from the online Alive-PD system except reminders to complete a 3-month and 6-month online follow-up questionnaire. Because participants had consented only to a 6-month delay before they could start the intervention, only the 3-month and 6-month results constitute the randomized trial portion of the study.

### The Alive-PD Intervention

**Figure 1:**
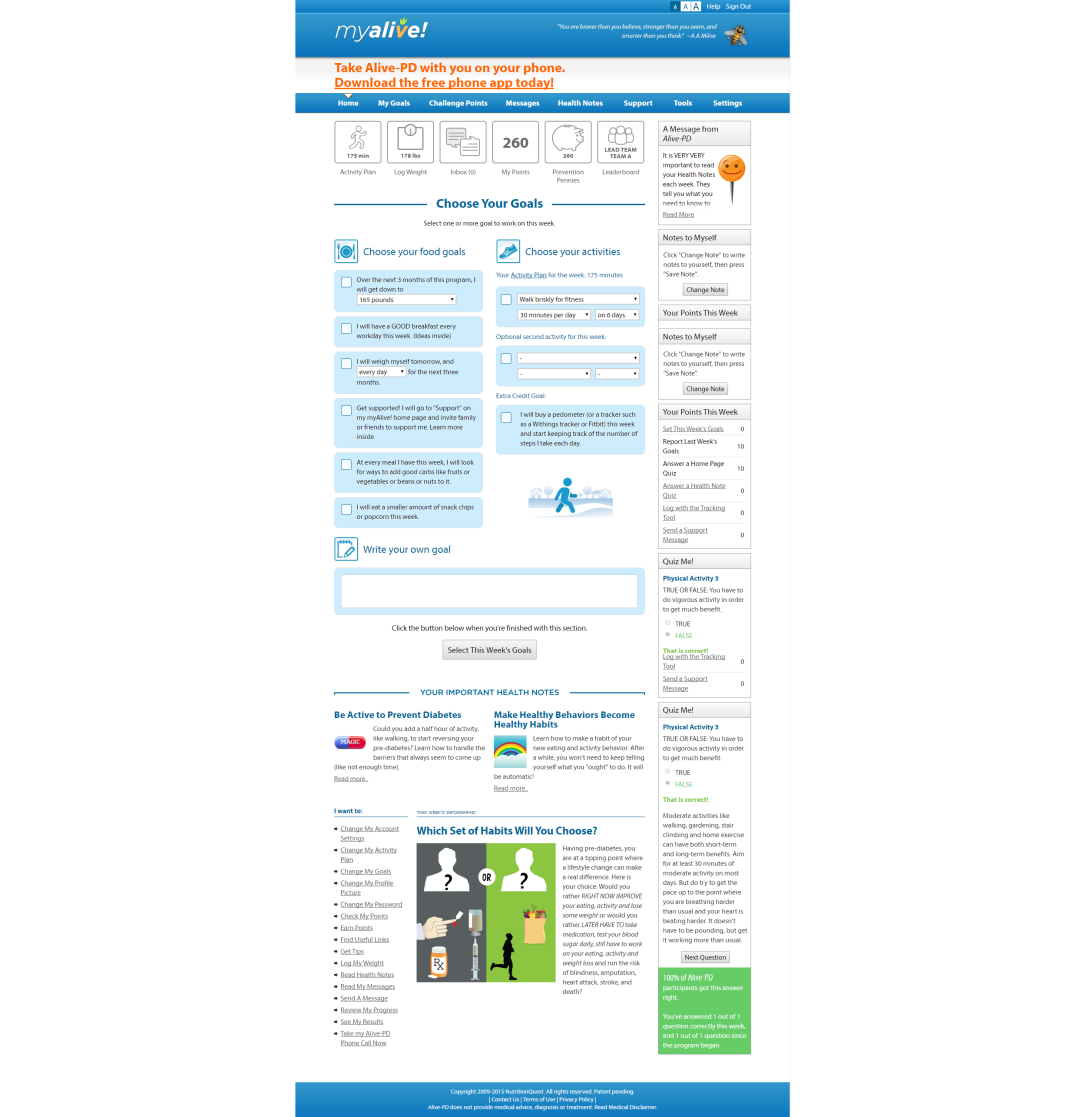
Screenshot of Alive-PD personal home page.

The program has been described in detail elsewhere [19]. Briefly, Alive-PD provides a 1-year program of regular contact and goal setting, weekly in the first 6 months and biweekly thereafter, plus midweek automated email and mobile phone reminders. The program includes individually tailored weekly goal setting and other activities delivered via Web and email supplemented by automated interactive voice response (IVR) phone calls and a supportive mobile phone app. Alive-PD was developed with input from, and was reviewed by, diabetes educators, endocrinologists, registered dietitians, and psychological experts in health behavior change. All features and contacts are completely automated and algorithm-driven, with no personal contact or coaching either in-person or remotely. See Figure 1 and Multimedia Appendix 2 for screenshots and other information.

The goal of the Alive-PD program is to improve glycemic control and reduce diabetes risk through lasting changes in physical activity and eating habits. Weight loss is encouraged and tracked as one of the changes that can reduce diabetes risk, although it is not the primary emphasis. For physical activity, participants set long-term goals of 150 to 300 minutes of aerobic activity per week depending on reported levels at baseline and on subsequent program participation. Resistance training is encouraged as well. For eating behaviors, the focus is on decreasing added sugars and refined carbohydrates, decreasing saturated and trans fats, and increasing fruit and vegetables. Changes in food type and reduction in portion size is emphasized as a means of reducing energy intake rather than specific calorie targets or counting. Psychosocial issues important in behavior change are addressed, including managing stress and sleep, staying motivated, addressing negative thoughts, modifying one’s environment to support desired changes, and other topics addressed in the DPP curriculum [21].

These objectives are achieved through a system of weekly individually tailored goal setting. Based on a detailed initial questionnaire on current dietary and activity habits, and on the participant’s subsequent interactions, the program recommends multiple weekly personally relevant small-step goals. Participants work on both increased physical activity and improved dietary habits each week, as well as occasional psychosocial goals. In addition to weekly personally tailored goals, the system provides tools for tracking weight, eating, and physical activity; weekly health information on diabetes and strategies for preventing it; quizzes; social support through virtual teams and a participant messaging system; feedback on reported diet and activity and on success or failure of goal achievement; weekly reminders; and other features. Engagement is promoted through a points system with modest monetary rewards and team competition. During the first 6 months, participants are reminded automatically if they have not chosen a goal for 2 weeks using data from the online system.

An email initiates the choice of weekly goals, provides a link to the participant’s Web page, and is followed up by a midweek reminder. An Android and iPhone app also permits the participant to select weekly goals, report on progress, and set mobile phone reminders. Automated motivational phone coaching is provided biweekly through IVR technology with interactions tailored to each individual’s participation status, barriers, and primary motivations.

These strategies and features are based on established principles derived from several bodies of behavior change research. The basic objective, derived from learning theory and other habit formation research [22-24], is to have participants gradually incorporate new eating and physical activity behaviors into their daily lives until these behaviors are both habitual and substantial enough to reduce diabetes risk. To accomplish that objective, a variety of strategies are employed to sustain involvement with the program itself and, more importantly, to sustain the gradual incorporation of new healthier behaviors. The strategies are consistent with several bodies of research, including models centering on cues and triggers [25,26], social cognitive theory [27,28], the theory of planned behavior [29], behavioral economics [26,30], and positive psychology [31,32]. For a more detailed description of the program, refer to the published protocol and program description [19].

### Subsequent Clinic Visits

Participants in the intervention and control groups returned for clinic visits at 3 and 6 months, at which time the laboratory and biometric measurements described previously were repeated by trained staff unaware of treatment assignment. Active monitoring of adverse events was achieved by asking participants about sickness or injury at each clinic visit. At the 6-month visit, additional funding made it possible to invite participants to continue the program for another 6 months, although the randomized trial segment ended at 6 months. Those in the intervention group continued in that arm. Those in the control group were transferred to the active Alive-PD intervention program per the original consent. Participants who consented to the extension were seen at additional clinic visits at 9 and 12 months.

### Statistical Methods

Intention-to-treat (ITT) analyses of change in HbA_1c_, fasting glucose, and weight were prespecified. Baseline characteristics were compared by chi-square tests for categorical variables and *t* tests for continuous variables. Mean between-group treatment differences in outcomes were evaluated by ITT analysis using linear regression approaches. In all models, change in the outcome of interest (eg, HbA_1c_) was the dependent variable with treatment group the main predictor (independent) variable and baseline value of the outcome variable as a covariate. Missing values in the dependent variable were imputed using the approach of Heckman et al [33,34], in which variables need not be assumed to be missing at random (MAR). This approach corrects for the bias in estimates of change that may arise from participants failing to complete the follow-up clinic visits. We examined potential interactions with treatment group by variables that were expected a priori to be potential effect modifiers (sex, race/ethnicity, age, and BMI category) by inclusion of a cross-product term in the model. No significant interactions were found. Adjustment for age, sex, BMI, and race/ethnicity did not materially alter the results. Dichotomous outcomes (eg, achievement of 5% weight loss) were evaluated by chi-square tests after confirming the absence of interactions using logistic regression. For comparability with other studies, we also conducted subgroup analyses on participants who were prediabetic by HbA_1c_ at baseline.

## Results

### Participant Randomization and Retention

A total of 340 participants met study eligibility criteria and were randomized. One participant randomized to the intervention group developed a metabolic condition rendering glycemic markers uninterpretable and was excluded from analysis, leaving 339 randomized participants.

Study retention and participation in biometric assessment visits was high; 89.1% (302/339) completed the 3-month follow-up assessment and 86.1% (292/339) completed the 6-month follow-up assessment. Of the 47 study participants that did not complete the 6-month follow-up (20 control, 27 intervention), 9 were lost to follow-up and 38 withdrew from the study. Reported adverse events were minor and all were considered to be unrelated to study participation. There were no significant differences in adverse events between treatment groups at either the 3-month or the 6-month visit (data not shown). One participant in the control group was diagnosed with diabetes and withdrew from the study; this participant did not provide follow-up measurements, but was included in the ITT analysis. No participants were prescribed metformin or other diabetes medications during the study.

### Baseline Characteristics

Participants were a mean age of 55 (SD 8.9, range 31-70) years with a mean BMI of 31.1 (SD 4.4) kg/m^2^ (Table 1). The majority (68.7%, 233/339) were male. Mean fasting glucose was at the low end of the prediabetic range (mean 6.1, SD 0.5 mmol/L or mean 109.9, SD 8.4 mg/dL) and mean HbA_1c_ was in the normal range (mean 5.6%, SD 0.3 or mean 38, SD 3.2 mmol/mol]). Metabolic syndrome was present in 68.1% (231/339) of participants. The study cohort was well educated; 82.9% (281/339) had a college degree or higher. The Framingham 8-year diabetes risk was 16.6% at baseline in both groups. The intervention and control groups were well balanced on baseline characteristics, although there was some imbalance for race/ethnicity, but it did not reach statistical significance (*P*=.07). This imbalance was due largely to a difference in Hispanic ethnicity (8.0%, 14/176 vs 4.3%, 7/163; *P*=.04). Due to this imbalance, race/ethnicity was examined for confounding and effect modification in all models.

**Table 1:** Baseline demographics and clinical characteristics.

Variable		All N=339	Control n=176	Intervention n=163	*P* ^a^
Age (years), mean (SD)		55.0 (8.9)	54.9 (9.1)	55.0 (8.8)	.88
Female, n (%)		106 (31.3)	54 (30.7)	52 (31.9)	.81
College or above, n (%)		281 (82.9)	144 (81.8)	137 (84.1)	.59
**Race/ethnicity, n (%)** ^b^					.07
	White	229 (67.6)	120 (68.2)	109 (66.9)	
	Hispanic	21 (6.2)	14 (8.0)	7 (4.3)	
	Asian	70 (20.6)	29 (16.5)	41 (25.2)	
	Other	19 (5.6)	13 (7.4)	6 (3.7)	
Metabolic syndrome, n (%)		231 (68.1)	121 (68.8)	110 (67.5)	.80
Weight (kg), mean (SD)		92.9 (15.8)	93.3 (16.6)	93.7 (14.9)	.68
BMI (kg/m^2^), mean (SD)		31.2 (4.4)	31.2 (4.3)	31.1 (4.5)	.73
Waist circumference (cm), mean (SD)		102.8 (10.8)	103.1 (11.2)	102.5 (10.4)	.62
Glucose (mmol/L), mean (SD)		6.10 (0.5)	6.08 (0.5)	6.11 (0.5)	.57
Glucose (mg/dL), mean (SD)		109.9 (8.4)	109.6 (8.3)	110.1 (8.6)	.57
HbA_1c_(%), mean (SD)		5.6 (0.3)	5.6 (0.3)	5.6 (0.3)	.90
HbA_1c_ (mmol/mol), mean (SD)		38.2 (3.2)	38.2 (3.1)	38.1 (3.3)	.90
Total cholesterol (mmol/L), mean (SD)		5.0 (0.8)	5.0 (0.9)	4.9 (0.8)	.82
LDL cholesterol (mmol/L), mean (SD)		3.0 (0.7)	3.0 (0.7)	3.0 (0.7)	.73
HDL cholesterol (mmol/L), mean (SD)		1.2 (0.4)	1.2 (0.3)	1.2 (0.4)	.34
Triglycerides (mmol/L), mean (SD)		1.6 (0.8)	1.7 (0.8)	1.6 (0.9)	.54
TG/HDL ratio, mean (SD)		3.5 (2.5)	3.6 (2.5)	3.4 (2.5)	.41
**Blood pressure (mm Hg), mean (SD)**					
	Systolic	130.4 (14.7)	130.4 (14.5)	130.5 (15.0)	.95
	Diastolic	82.3 (8.4)	82.6 (8.7)	82.0 (8.1)	.51
Framingham 8-year diabetes risk (%), mean (SD)		16.63 (10.67)	16.64 (10.78)	16.63 (10.58)	.99

^a^ Significance of difference between intervention and control.

^b^ Race and ethnicity as reported on online questionnaire. Native American/Alaskan, Native Hawaiian/Pacific Islander, more than one race, or “not reported” reported as “other.”

### Primary Outcomes

**Figure 2:**
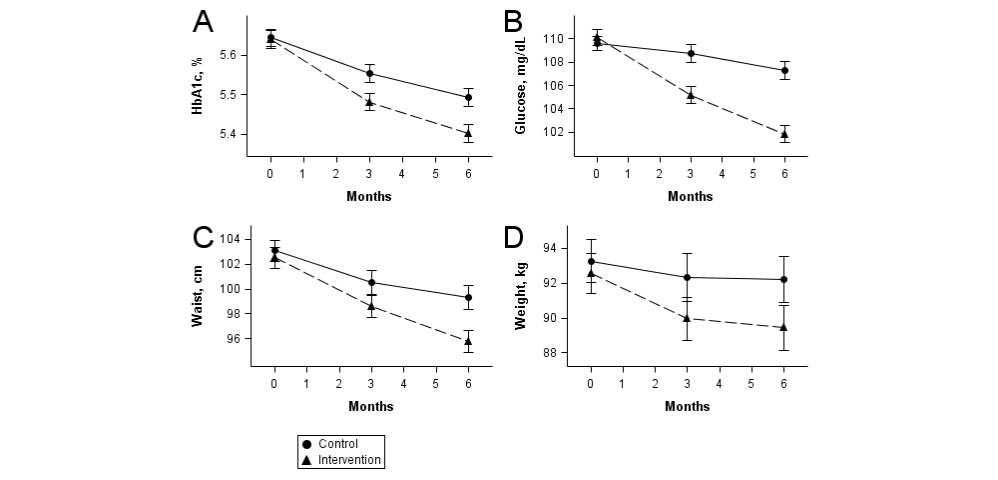
Changes in primary and secondary endpoints over time. Solid line: control; dashed line: intervention; error bars: ± standard error. A: Change in HbA1c. B: Change in fasting glucose. C: Change in waist circumference. D: Change in weight. At 6 months, all measures were significantly different between control and intervention groups (*P*<.001).

Significant decreases in HbA_1c_ and fasting glucose were observed in the intervention group by 3 months from baseline and declined further at 6 months (Figure 2).

In ITT analyses, which included all 339 participants, mean reductions in fasting glucose at 6 months from baseline were significantly greater in the intervention group (mean –0.41 mmol/L, 95% CI –0.44 to –0.12) than in the control group (mean –0.21 mmol/L, 95% CI –0.15 to –0.10; *P*<.001) (Table 2). Mean reductions in HbA_1c_ were also significantly greater in the intervention versus the control group (mean –0.26%, 95% CI –0.27 to –0.24 vs mean –0.18%, 95% CI –0.19 to –0.16; *P*<.001). No effect modification by race/ethnicity, age, sex, or BMI category was observed.

Although all participants had prediabetes at baseline by either HbA_1c_ or fasting glucose, only 44.8% (152/339) had prediabetes based on HbA_1c_. In a subgroup analysis among those with prediabetes at baseline by HbA_1c_ (Table 2), the mean reduction in HbA_1c_ was greater than in the intervention group as a whole (mean –0.32%, 95% CI –0.38 to –0.26) and was significantly greater relative to the control group (mean –0.20%, 95% CI –0.25 to –0.15; *P*=.002).

**Table 2:** Change in clinical outcomes by treatment group.

Variable	Intention-to-treat,^a^ change (95% CI)^b^			Prediabetic by HbA_1c_,^c^ change (95% CI)^b^		
	Alive-PD n=163	Control n=176	*P*	Alive-PD n=60	Control n=69	*P*
Fasting glucose (mg/dL)	–7.36 (–7.85, –6.87)	–2.19 (–2.64, –1.73)	<.001	–7.38 (–9.40, –5.36)	–1.23 (–3.12, 0.65)	<.001
Fasting glucose (mmol/L)	–0.41 (–0.44, –0.38)	–0.12 (–0.15, –0.10)	<.001	–0.41 (–0.52, –0.30)	–0.07 (–0.17, 0.04)	<.001
HbA_1c_ (%)	–0.26 (–0.27, –0.24)	–0.18 (–0.19, –0.16)	<.001	–0.32 (–0.38, –0.27)	–0.20 (–0.25, –0.15)	.002
HbA_1c_ (mmol/mol)	–2.81 (–2.95, –2.66)	–1.93 (–2.06, –1.79)	<.001	–3.50 (–4.10, –2.90)	–2.15 (–2.71, –1.59)	.002
Weight (kg)	–3.26 (–3.26, –3.25)	–1.26 (–1.27, –1.26)	<.001	–3.56 (–4.42, –2.70)	–0.48 (–1.28, 0.32)	<.001
Weight loss (%)	–3.60 (–3.63, –3.57)	–1.32 (–1.36, –1.28)	<.001	–4.00 (–4.94, –3.07)	–0.53 (–1.40, 0.34)	<.001
BMI (kg/m^2^)	–1.05 (–1.06, –1.05)	–0.39 (–0.39, –0.38)	<.001	–1.19 (–1.47, –0.90)	–0.17 (–0.43, 0.10)	<.001
Waist (cm)	–4.56 (–4.69, –4.43)	–2.22 (–2.36, –2.09)	<.001	–7.23 (–8.99, –5.47)	–2.73 (–4.37, –1.10)	<.001
TG/HDL ratio	–0.21 (–0.30, –0.12)	0.21 (0.12,0.29)	.04	–0.43 (–0.85, –0.02)	0.12 (–0.27, 0.51)	.06

^a^ Imputation of missing dependent variables using Heckman/QLIM.

^b^ 95% confidence limits from least squares means from models of following form: change=baseline + treatment group.

^c^ Participants prediabetic by HbA_1c_ at baseline and providing complete data.

### Secondary Outcomes

In the ITT analysis, reduction in weight, BMI, waist circumference, and TG/HDL ratio were all significantly greater in the intervention group than the control group (Table 2). The intervention group lost a mean 3.26 kg (95% CI –3.26 to –3.25) compared to 1.26 kg (95% CI –1.27 to –1.26) in the control group (*P*<.001). Mean BMI was reduced by 1.05 kg/m^2^ (95% CI –1.06 to –1.05) and 0.39 kg/m^2^ (95% CI –0.39 to –0.38) in the intervention and control groups, respectively (*P*<.001). The mean reduction in waist circumference in the intervention group was 4.56 cm (95% CI –4.69 to –4.43) compared to 2.22 cm (95% CI –2.36 to –2.09) in the control group (*P*<.001). In addition, the ratio of TG/HDL was significantly reduced in the intervention group in contrast to the increase seen in the control group (mean –0.21, 95% CI –0.30 to –0.12 vs mean 0.21, 95% CI 0.12-0.29; *P*=.04).

**Figure 3:**
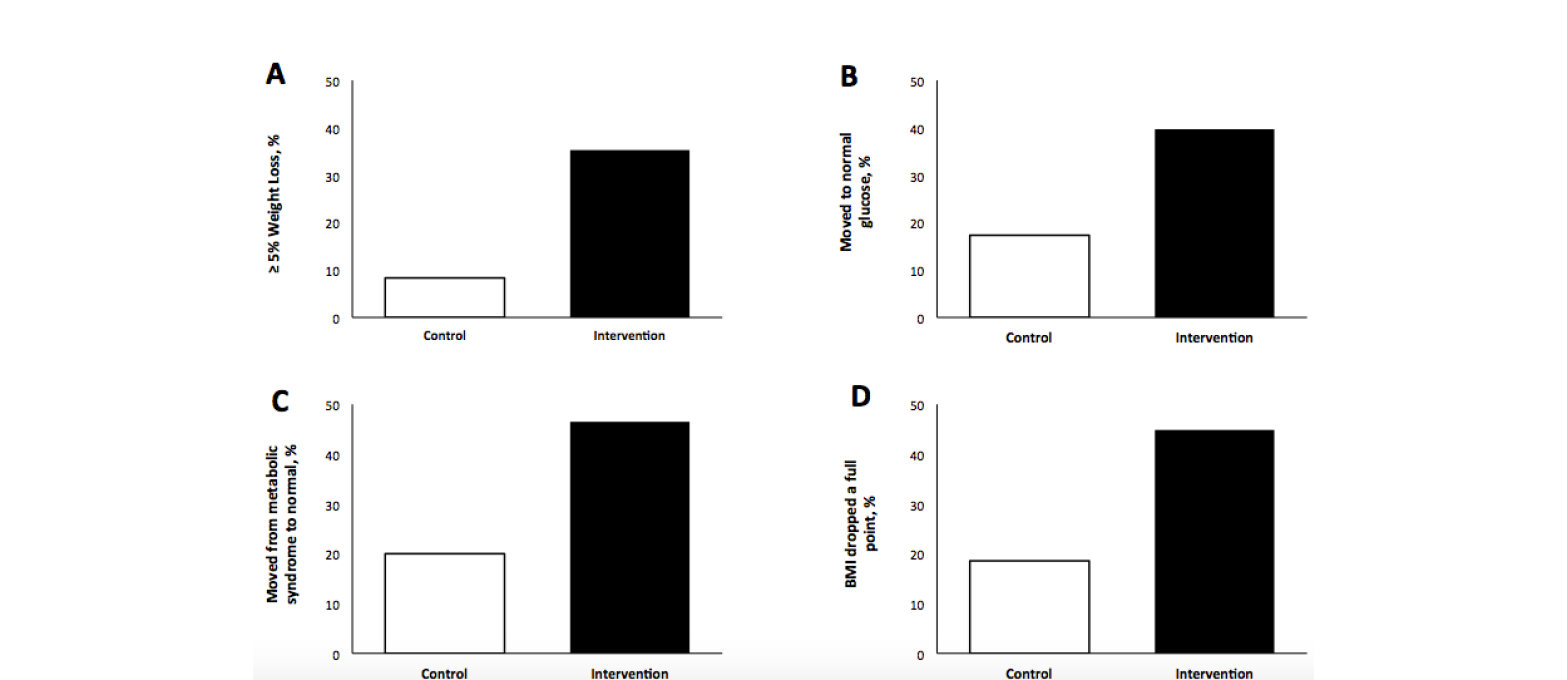
Proportion achieving secondary endpoint thresholds at 6 months. Error bars not shown because all differences between control and intervention were *P*<.001. A: Percentage with ≥5% weight loss (complete data: n=156 control, n=136 intervention). B: Percentage who moved to normal fasting glucose (from ≥100 mg/dL to <100 mg/dL) (denominator: n=150 control, n=126 intervention). C: Percentage who moved from having metabolic syndrome to not having metabolic syndrome (denominator: n=110 control, n=86 intervention). D: Percentage whose BMI decreased by 1 kg/m^2^ (denominator: n=156 control, n=136 intervention).

The proportion of participants, by treatment group, meeting specific thresholds are shown in Figure 3. At 6 months, 35.3% (48/136) of the intervention group had achieved at least a 5% weight loss compared to 8.3% (13/156) of controls (Figure 3A). Among those who were prediabetic by fasting glucose at baseline, 40.5% (49/121) of intervention participants had achieved a normal fasting glucose compared to 17.7% (26/147) of controls (Figure 3B). Among participants who had metabolic syndrome at baseline, 46.5% (40/86) of those in the intervention group no longer had metabolic syndrome at 6 months compared with 20.0% (22/110) of controls (Figure 3C). BMI was reduced by at least 1 kg/m^2^ in 44.9% (61/136) of intervention participants compared with 18.6% (29/156) of control participants (Figure 3D). All these differences between the intervention and control groups were significant at *P*<.001.

**Figure 4:**
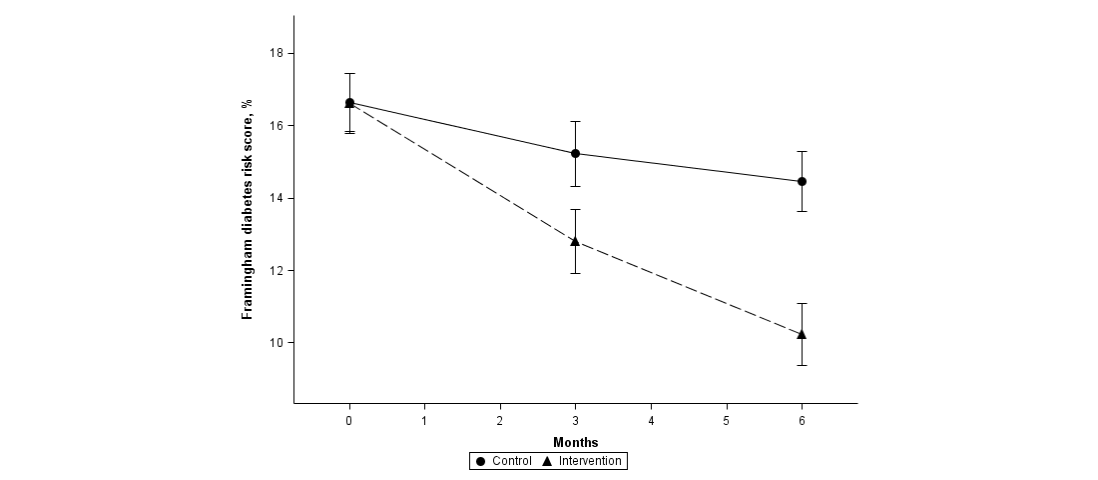
Change in Framingham 8-year diabetes risk.

There was a significantly greater reduction in Framingham 8-year diabetes risk in the intervention versus the control group (*P*<.001) in the ITT sample (Figure 4). In both groups, the baseline diabetes risk was 16%. At 6 months, it was 11.00% (95% CI 10.08-11.92) in the intervention group and 14.59% (95% CI 13.64-15.54) in the control group.

### Case Report on Participants in the Diabetic Range by Fasting Glucose

Alive-PD was designed to assist persons with prediabetes. However, lifestyle behavior change is also an essential intervention for persons who are newly diagnosed with diabetes. Thus, information about results in the 8 participants in our sample who had a fasting glucose in the diabetes range at baseline is of interest (they were all cleared by their physicians for participation in the study). Of the 5 in the intervention group, one had a decrease in fasting glucose to the normal range (<100 mg/dL) and the other 4 had a decrease in fasting glucose to the prediabetic range (<126 mg/dL) after the 6-month intervention period. None of the 3 participants in the control group had decreases in glucose outside of the diabetic range.

### Process Measures and Other Behaviors

We assessed program participation by evaluating the points each participant earned through interacting with the program components each week and by assessing the participants’ weekly goal setting behaviors. Participation in the online Alive-PD program features was high. Intervention participants (ITT population, n=163) set behavioral goals or otherwise interacted with the online Alive-PD program in a median of 17 (IQR 14) of the 24 weeks (70.8% of the weeks). In all, 87.1% (142/163) interacted with the program in 4 or more of the 24 weeks and 70.6% (115/163) were still interacting with the program in the last month of the 6-month period. Participants accomplished a median of 35 goals (IQR 107) in the 24-week period or approximately 1.5 goals per week. Intervention participants reported that they spent approximately 15 minutes interacting with the program in a typical week.

The intervention group experienced significant improvements in self-reported physical activity, dietary habits, sleep, fatigue, and self-confidence relative to the control group (*P*<.001) (data not shown). A more detailed analysis of changes in physical activity, diet, self-confidence, and other psychosocial factors will be reported elsewhere.

## Discussion

In this randomized controlled trial, the fully automated Alive-PD program was effective in improving glycemic control and body weight, and in reducing 8-year diabetes risk. In ITT analyses, the intervention group achieved reductions in fasting glucose of –41 mmol/L (–7.36 mg/dL) and in HbA_1c_ of –0.26% (–3 mmol/mol), both statistically significantly superior to changes in the control group. In addition, intervention group participants lost a mean 3.26 kg over 6 months, in ITT analyses, and 35% of the intervention group lost 5% or more of initial body weight, both significantly superior to the control group.

### Previous Research on Weight Loss in Diabetes Prevention or Weight Loss Programs

Numerous reviews of weight loss or translational diabetes prevention programs have been conducted [9,12,35-42] covering a range of delivery methods. Interventions using in-person or group approaches have achieved average weight losses of approximately 3% to 4% in reviews and meta-analyses [9,37], although some individual studies have reached weight losses of more than 6% in the intervention group [6,43].

For wider reach, however, many interventions have combined coaches with some form of technology. In a 2015 review of 16 studies of technology-assisted programs for weight loss in primary care, Levine et al [12] found a median weight loss of -2.7 kg in intervention groups of 12 programs that combined human with technological methods. Ali et al [9] found a mean loss of 4.2% of body weight in electronic media-assisted programs.

Interventions delivered entirely by electronic media, primarily for weight loss, have also been reviewed. Hartmann-Boyce et al [13] conducted a meta-analysis of 23 randomized trials of “self-help interventions” for weight loss in overweight or obese adults. Programs were not eligible for inclusion if they used any form of person-to-person assistance by counselors or health professionals. The analysis found a mean difference between intervention and comparison groups of -1.85 kg (95% CI -2.86 to -0.83) at 6 months. Three programs using eHealth technologies that were not included in the Hartmann-Boyce review were found by Hutchesson et al [14] to have a mean difference of -1.5 kg. Three other fully automated studies from the Levine review [12] found a mean weight loss in the intervention group of 2.5 kg. One recent trial not included in previous reviews [44] was fully automated with the exception of a 60-minute baseline visit at which participants were given weight loss, calorie and physical activity goals, and taught behavioral skills. A weight loss of 5.4 kg was observed at 6 months.

The effect of Internet-based interventions on change in waist circumference has also been examined in a meta-analysis. Seo and Niu [45] found a mean change of -2.99 cm (95% CI −3.68 to −2.30).

### Previous Research on the Effect of Fully Automated Programs on Glycemic Markers

With few exceptions, most studies of diabetes prevention or weight loss interventions using fully automated programs have not measured or reported on changes in glycemic markers. One review found “minimal” changes in glycemic markers across the reviewed studies, with a median change in fasting glucose of -0.2 mmol/L [37] and another found a mean change of -0.1 mmol/L [38]. For HbA_1c_, Dunkley et al [38] found pooled changes of -0.13% and Johnson et al [37] found a median change of -0.05%.

The treatment effects for Turnaround Health’s Alive-PD program are consistent with and, in most cases, somewhat larger than the results summarized in the preceding meta-analyses. This is true for weight loss (–3.26 kg), percent weight loss (–3.60%), waist circumference (–4.56 cm), and the glycemic markers HbA_1c_ (–0.26%) and fasting glucose (–0.41 mmol/L), all in ITT analyses.

### The Diabetes Prevention Recognition Program

The Centers for Disease Control and Prevention (CDC) Diabetes Prevention Recognition Program (DPRP) is intended to recognize organizations that have demonstrated their ability to deliver a proven type 2 diabetes prevention lifestyle intervention [46]. The CDC recently updated the requirements for recognition to include programs delivered “virtually” provided they meet other criteria. Turnaround Health’s Alive-PD program is listed on the CDC website [47]. As of August 1, 2015, it is the only such program with evidence of effectiveness from a randomized controlled trial and the only study with ITT analysis.

### Features Promoting Effectiveness

A number of authors have attempted to identify or summarize what features of a behavioral intervention may be associated with its effectiveness [40,42,48]. The following have all been identified as contributors to effectiveness: goal setting, self-monitoring, tailoring and tailored feedback, reminders, social support, and a structured program employing behavior change principles. Khaylis et al [48] also listed feedback by a counselor as an important feature, but noted that computer-automated email feedback has been as effective as human email counseling in at least one study. With the exception of human counseling, all these features are incorporated into the Alive-PD program. In addition, Alive-PD added some gamification features, such as a points system, team competition, and monetary rewards, to enhance engagement and retention.

Research is underway to explore which features of Alive-PD may be more important or beneficial. Although all participants were exposed to all these components (goal setting, messaging, etc), different participants engaged in them to different extents. For example, 38.7% (63/163) never logged their weight or physical activity, whereas 12.3% (20/163) logged their weight or activity in 21 or more of the 24 weeks. Mediation analyses are underway. However, it is worth noting that participants are individuals with varying interests and motivations. Some people appreciate being on a team whereas others dislike it and the same can be said of other components. Alive-PD was intentionally designed to provide an array of components to engage the widest range of different interests, learning styles, and available time.

In addition to the potential role of features of an intervention, it is also of considerable interest to explore what behaviors and specific changes contributed to the study outcomes. Recent literature has discussed the relative roles of types of macronutrients (fats vs carbohydrates), physical activity, and weight loss [49-51]. The Alive-PD program promoted, and achieved, increases in physical activity, reductions in refined carbohydrates, reductions in saturated and trans fats, and increases in fruits and vegetables. Changes in specific foods were also encouraged, such as nuts, legumes, and olive oil. Participants in the intervention group undertook these changes to varying degrees. In future analyses, we will examine the effect of these variations on changes in glycemic markers and weight. For example, there was a significant reduction in HbA_1c_, even among those who did not achieve 5% weight loss. We plan to explore factors that contributed to glycemic improvements in the absence of major weight loss.

### Limitations

The fully automated nature of the Alive-PD program is both a strength and a limitation. Some people need and respond better to human interaction and support, and effect sizes might be greater if combined with human support. In addition, because the intervention is delivered by email, Internet, and mobile phone, it may have limited reach for those who do not have Internet access or who are not technologically proficient. Although its reach is somewhat limited in that respect, 87% of American adults used the Internet as of 2014, including more than 80% of African Americans and Hispanics [52]. These technologies are nearly ubiquitous in society and allow for convenient program access at home or through mobile devices. At the same time, the fully automated characteristic of the program is beneficial for several reasons. There is a guarantee of 100% fidelity to the design and content in future administrations and enhancements can be readily incorporated. Because it is fully automated, this commercial program can be delivered at low cost and with wide reach. Additionally, organizations using it would require no or minimal staff.

Although the Alive-PD program provides a 1-year intervention, the randomized trial analysis was for only a 6-month period. This was due to initial funding limitations and the desire to enhance enrollment of these persons at high risk of developing diabetes by assuring them that they would be given access to the active program in a reasonable period of time. It will be important to follow study participants for a full year to determine whether the trends seen in Figure 2 are maintained.

Study participants were relatively well educated and two-thirds were non-Hispanic white. Thus, the generalizability to less educated individuals and those of race/ethnic minority groups remains to be investigated. However, it is notable that the subgroup with postgraduate or professional degrees achieved less improvement in glycemic markers than those with lower educational levels (data not shown.) The sample did include a substantial number of Asians (21% of the study cohort) including South Asians, a group for which type 2 diabetes is especially common. Although analyses indicated no significant differences in treatment effects by ethnicity, more research is needed to confirm effectiveness in minority groups.

### Clinical Relevance

The decrease in fasting glucose in the intervention group (–7.36 mg/dL or –0.41 mmol/L) was clinically meaningful and substantial. The decrease in HbA_1c_ was modest (–0.26% or –2.81 mmol/mol in the ITT analysis and –0.32% or –3.5 mmol/mol in those prediabetic by HbA_1c_), but significantly greater than in controls. We note that baseline levels of HbA_1c_ were low in the study cohort. Indeed, mean HbA_1c_ at baseline was in the normal range and only 45% were prediabetic by the HbA_1c_ definition. As a result, the magnitude of the average treatment effect was not as large as might be expected in patients with higher values in the prediabetic range or in those with diabetes. Weight loss was 4% of baseline weight among those prediabetic by HbA_1c_ (Table 2) and increased with increasing participation in the program and higher baseline weight (data not shown). As noted, the primary objective was to lower glycemic markers, a direct measure of reduced diabetes risk, and this appears to have been achieved despite the relatively modest weight loss. The Alive-PD group’s decreases in HbA_1c_ and fasting glucose were greater than those seen in the Diabetes Prevention Program [5] Lifestyle group at 6 months (HbA_1c_: -0.26% vs -0.09%; fasting glucose: -7.36 mg/dL vs 4.59 mg/dL, respectively), despite the fact that the Alive-PD group’s weight loss was not as great as that seen in DPP (3.26 kg vs -6.5 kg) [5].

More than two-thirds of enrollees were male, a different sex distribution than is usually seen in health interventions (the DPP had 68% female participants) [5]. The electronic format may have had more appeal for men than a series of group or personal interactions. There was not a significant interaction between sex and treatment effect, and treatment effects were not significantly different by sex for HbA_1c_, fasting glucose, or weight.

### Summary

In summary, Alive-PD was effective in improving markers of glycemic control and body weight in patients with prediabetes. As noted by Cefalu et al [4], the driving force behind the increased economic burden of diabetes is increased prevalence. Therefore, engaging as many as possible of the nation’s 86 million adults with prediabetes with a variety of cost-effective interventions is an urgent priority. Effective fully automated technologies such as Alive-PD represent one of those strategies, with the potential of serving large numbers of persons at risk of progression to diabetes.

## Appendix

Multimedia Appendix 1 CONSORT-EHEALTH checklist.

Multimedia Appendix 2 Powerpoint presentation with screenshots of Alive-PD.

## Abbreviations

BMI: body mass index
CDC: Centers for Disease Control and Prevention
DPP: Diabetes Prevention Program
DPRP: Diabetes Prevention Recognition Program
HDL: high-density lipoprotein
ITT: intention-to-treat
IVR: interactive voice response
PAMF: Palo Alto Medical Foundation
TG: triglyceride
