# Supplementary material for: Diabetes Prevention and Weight Loss with a Fully Automated Behavioral Intervention by Email, Web, and Mobile Phone: A Randomized Controlled Trial Among Persons with Prediabetes
Source: J Med Internet Res. 2015 Oct 23;17(10):e240. doi: 10.2196/jmir.4897 (PMC4642405; doi:10.2196/jmir.4897)
Supplement: Multimedia Appendix 2 [file jmir_v17i10e240_app2.ppt]

## Slide 1
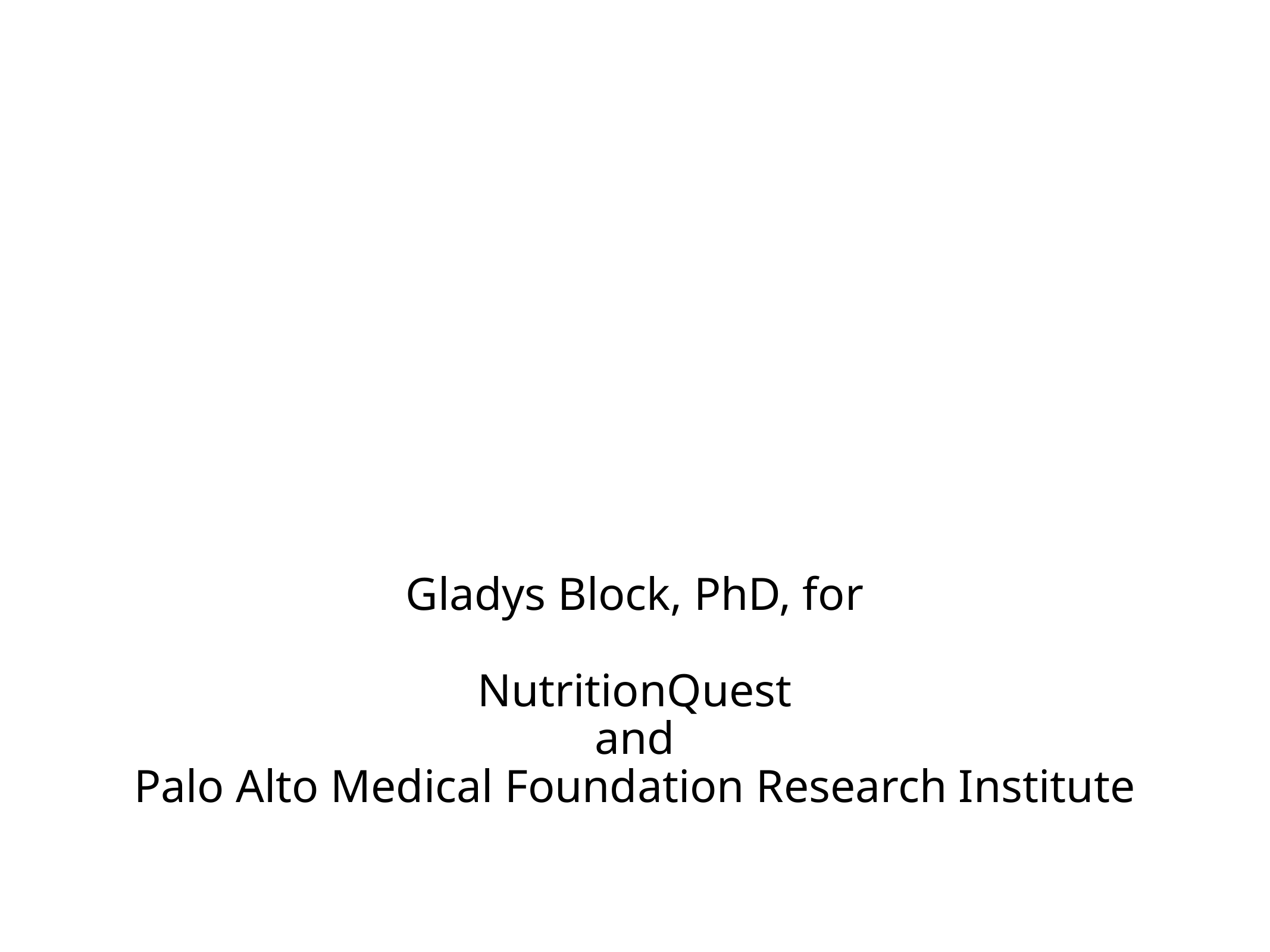

# Randomized Controlled Trial of a Web- and Internet-Based Diabetes Prevention Program Alive-PD
Gladys Block, PhD, for
NutritionQuest
and
Palo Alto Medical Foundation Research Institute

## Slide 2
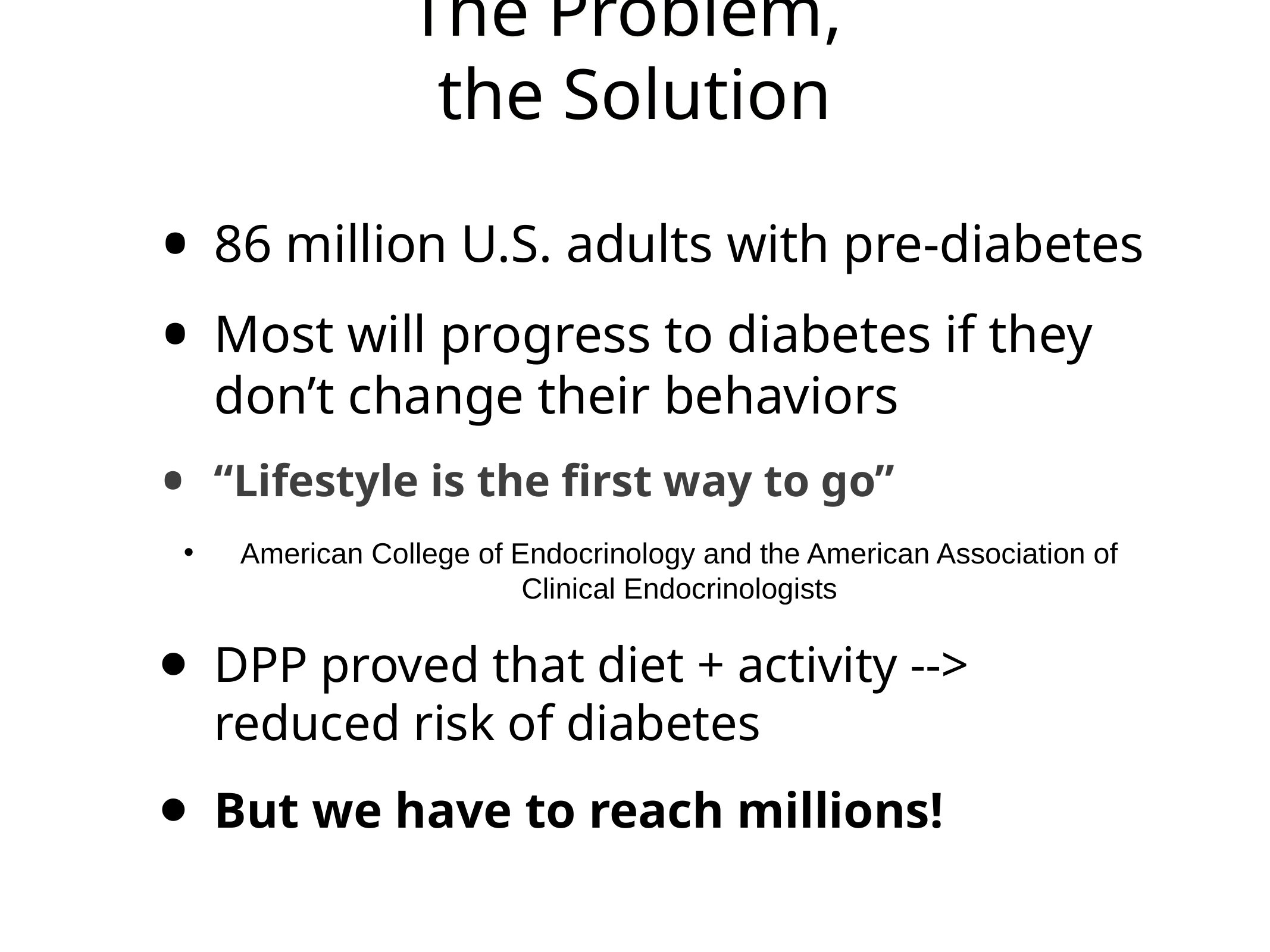

# The Problem, the Solution
86 million U.S. adults with pre-diabetes
Most will progress to diabetes if they don’t change their behaviors
“Lifestyle is the first way to go”
American College of Endocrinology and the American Association of Clinical Endocrinologists
DPP proved that diet + activity --> reduced risk of diabetes
But we have to reach millions!

## Slide 3
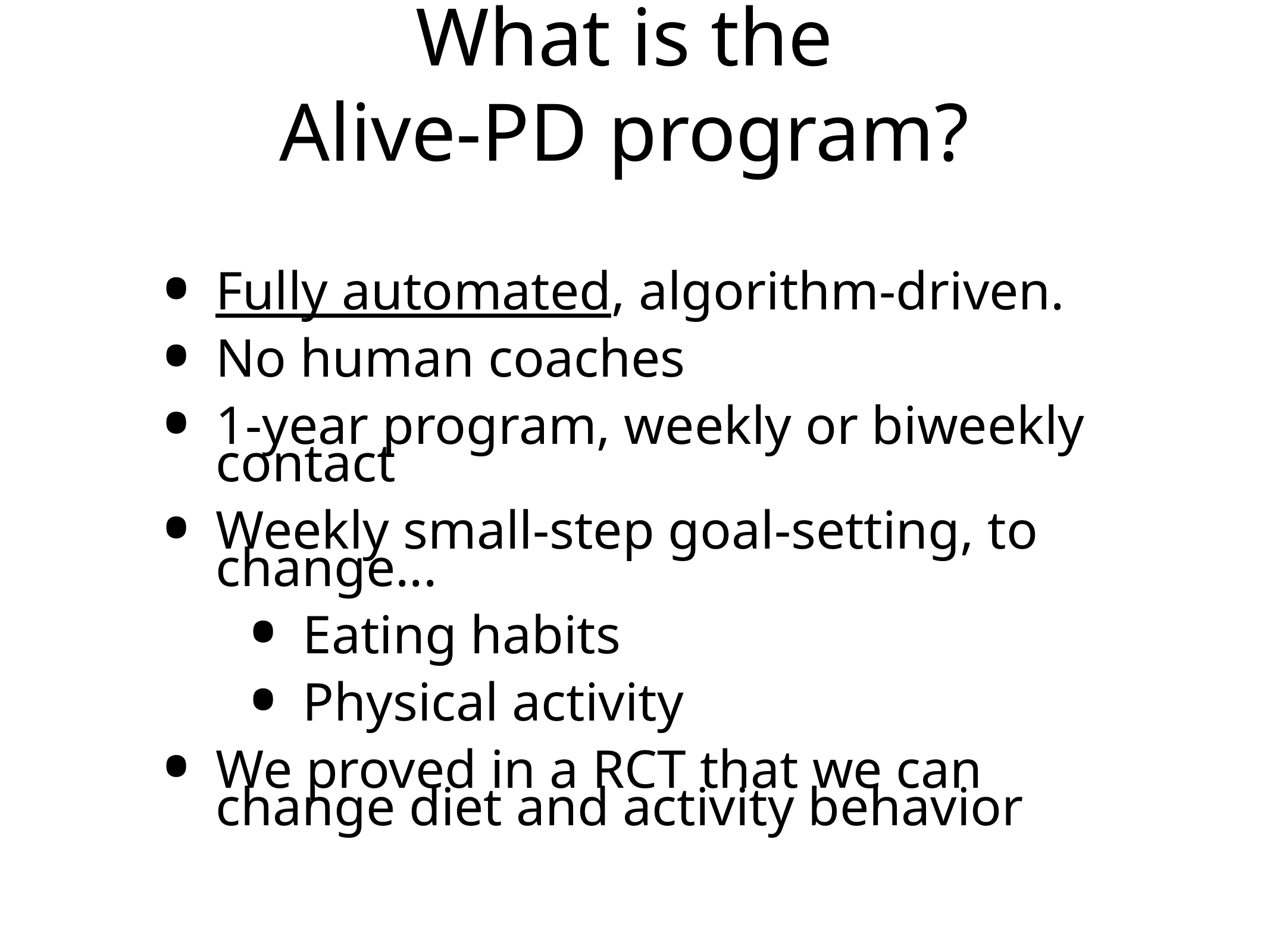

# What is the Alive-PD program?
Fully automated, algorithm-driven.
No human coaches
1-year program, weekly or biweekly contact
Weekly small-step goal-setting, to change...
Eating habits
Physical activity
We proved in a RCT that we can change diet and activity behavior

## Slide 4
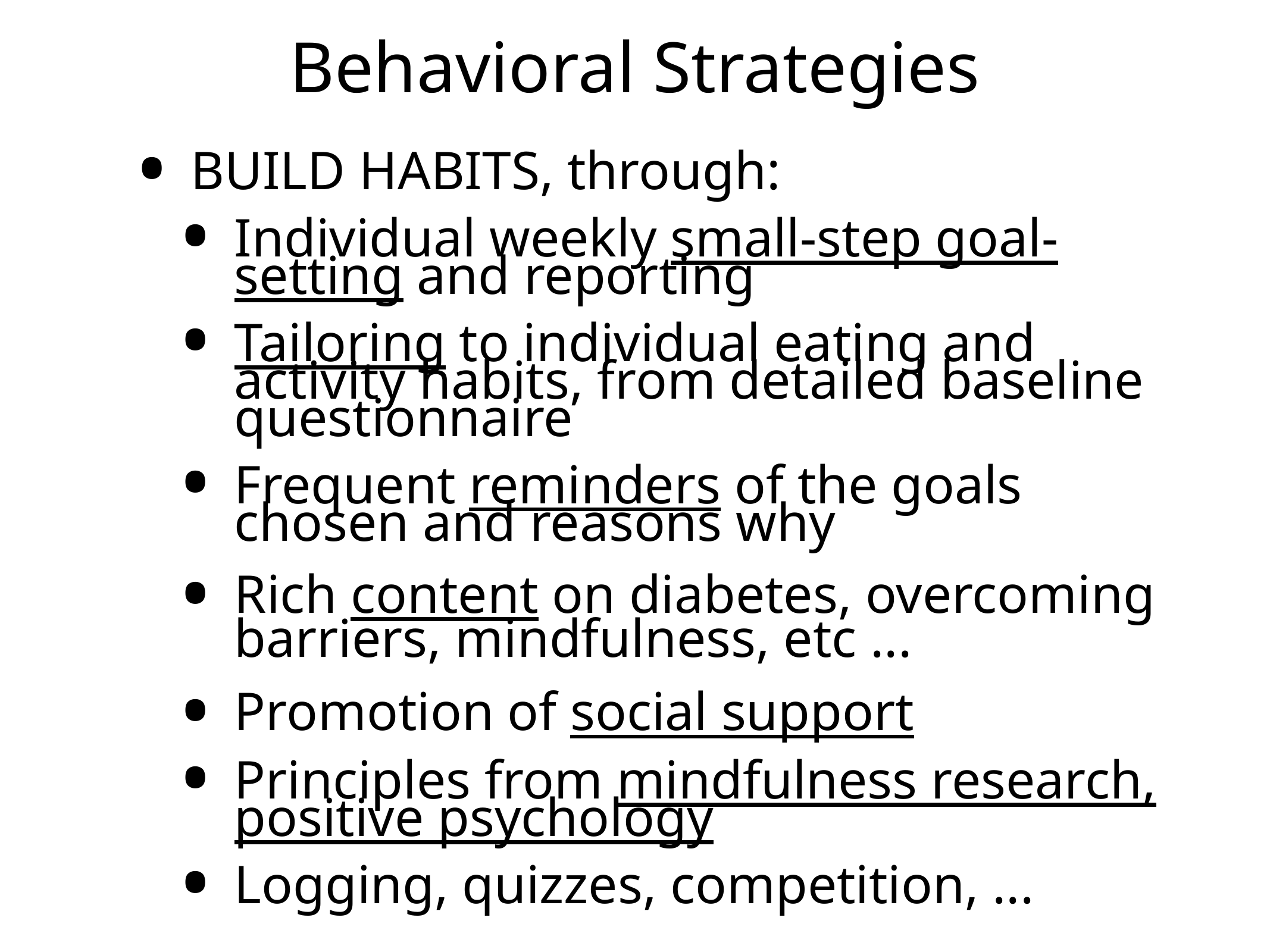

# Behavioral Strategies
BUILD HABITS, through:
Individual weekly small-step goal-setting and reporting
Tailoring to individual eating and activity habits, from detailed baseline questionnaire
Frequent reminders of the goals chosen and reasons why
Rich content on diabetes, overcoming barriers, mindfulness, etc ...
Promotion of social support
Principles from mindfulness research, positive psychology
Logging, quizzes, competition, ...

## Slide 5
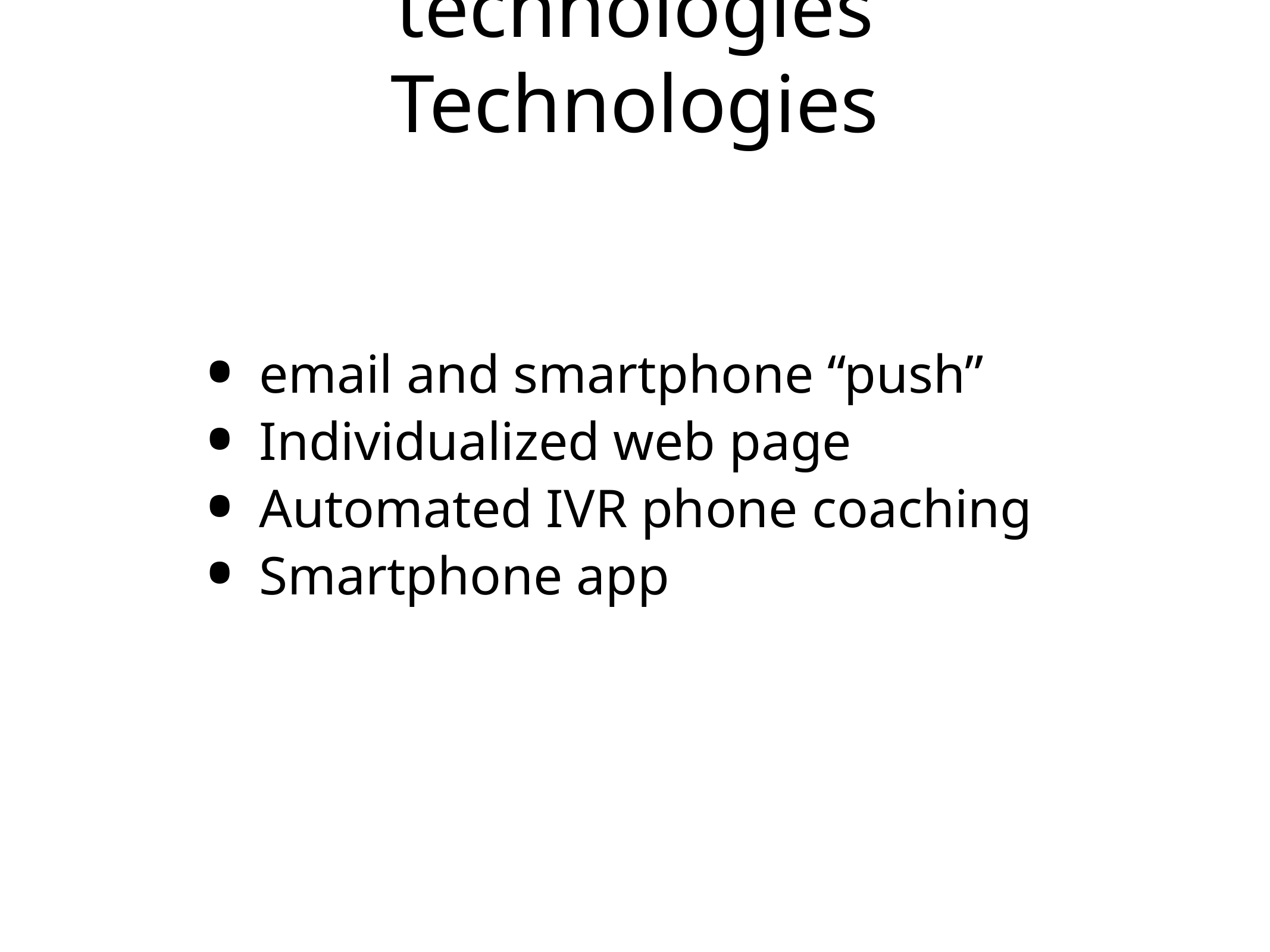

# Alive-PD program technologies Technologies
email and smartphone “push”
Individualized web page
Automated IVR phone coaching
Smartphone app

## Slide 6
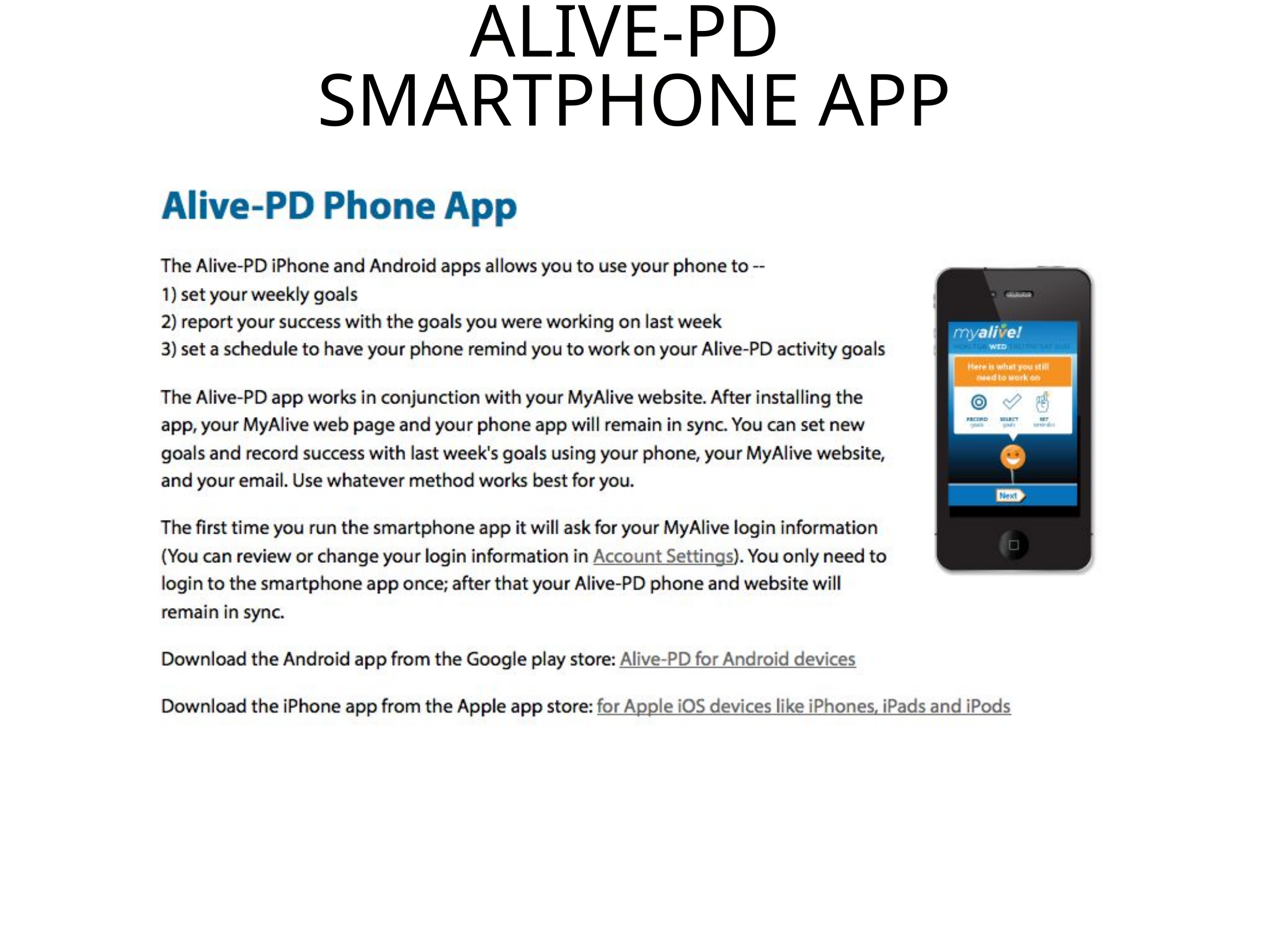

# ALIVE-PD SMARTPHONE APP

## Slide 7
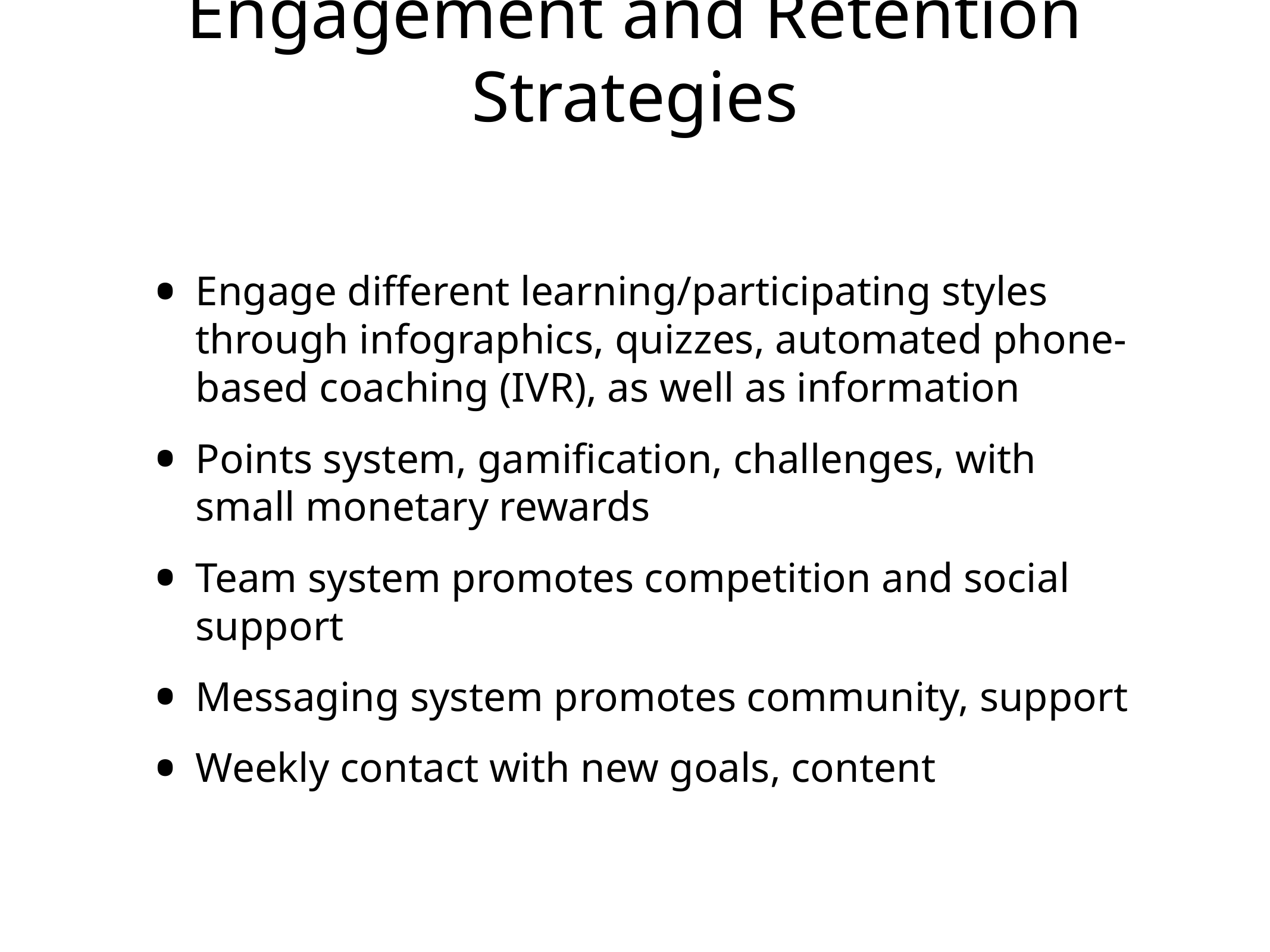

# Engagement and Retention Strategies
Engage different learning/participating styles through infographics, quizzes, automated phone-based coaching (IVR), as well as information
Points system, gamification, challenges, with small monetary rewards
Team system promotes competition and social support
Messaging system promotes community, support
Weekly contact with new goals, content

## Slide 8
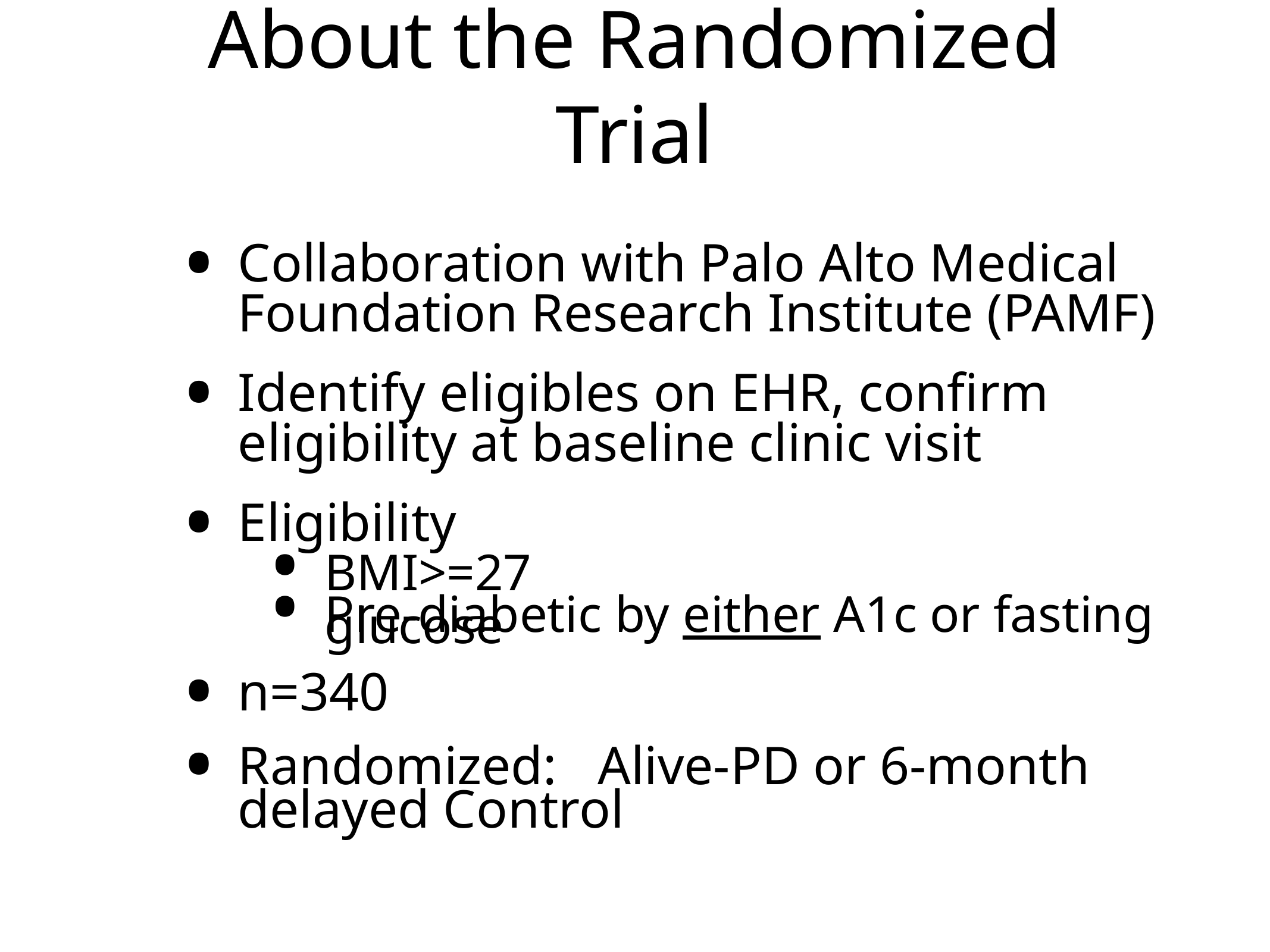

# About the Randomized Trial
Collaboration with Palo Alto Medical Foundation Research Institute (PAMF)
Identify eligibles on EHR, confirm eligibility at baseline clinic visit
Eligibility
BMI>=27
Pre-diabetic by either A1c or fasting glucose
n=340
Randomized: Alive-PD or 6-month delayed Control

## Slide 9
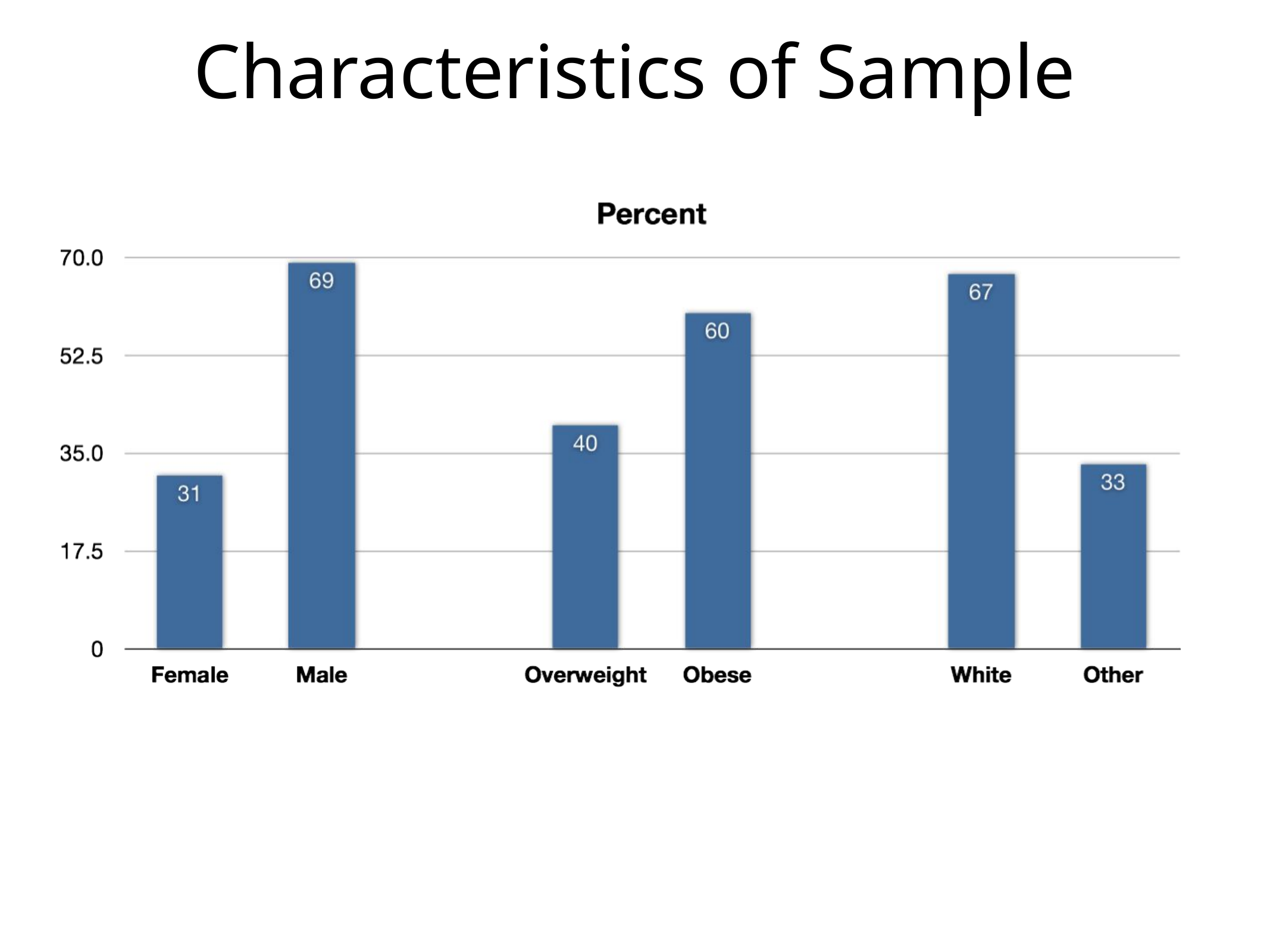

# Characteristics of Sample

## Slide 10
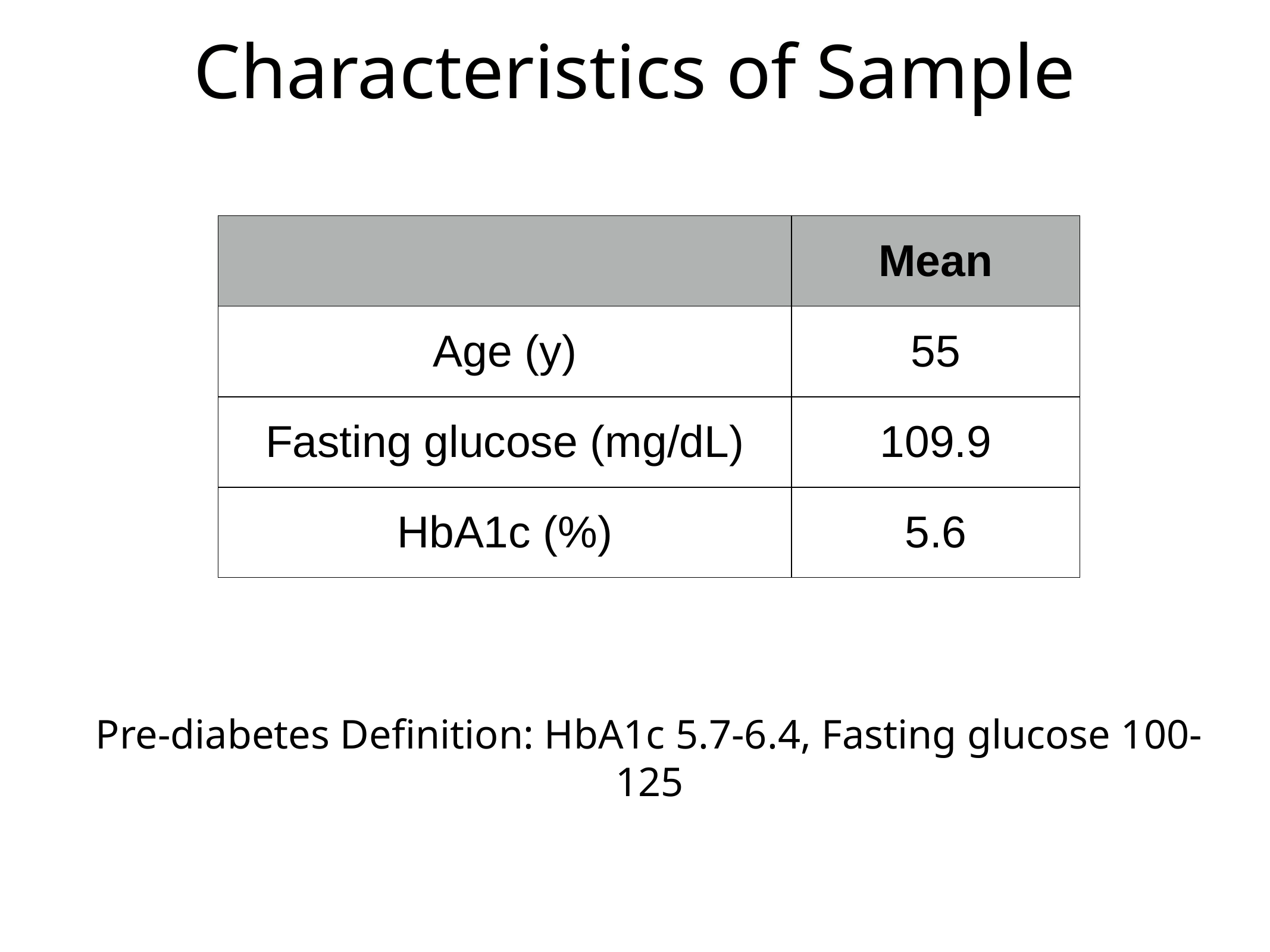

# Characteristics of Sample
| | Mean |
| --- | --- |
| Age (y) | 55 |
| Fasting glucose (mg/dL) | 109.9 |
| HbA1c (%) | 5.6 |
Pre-diabetes Definition: HbA1c 5.7-6.4, Fasting glucose 100-125

## Slide 11
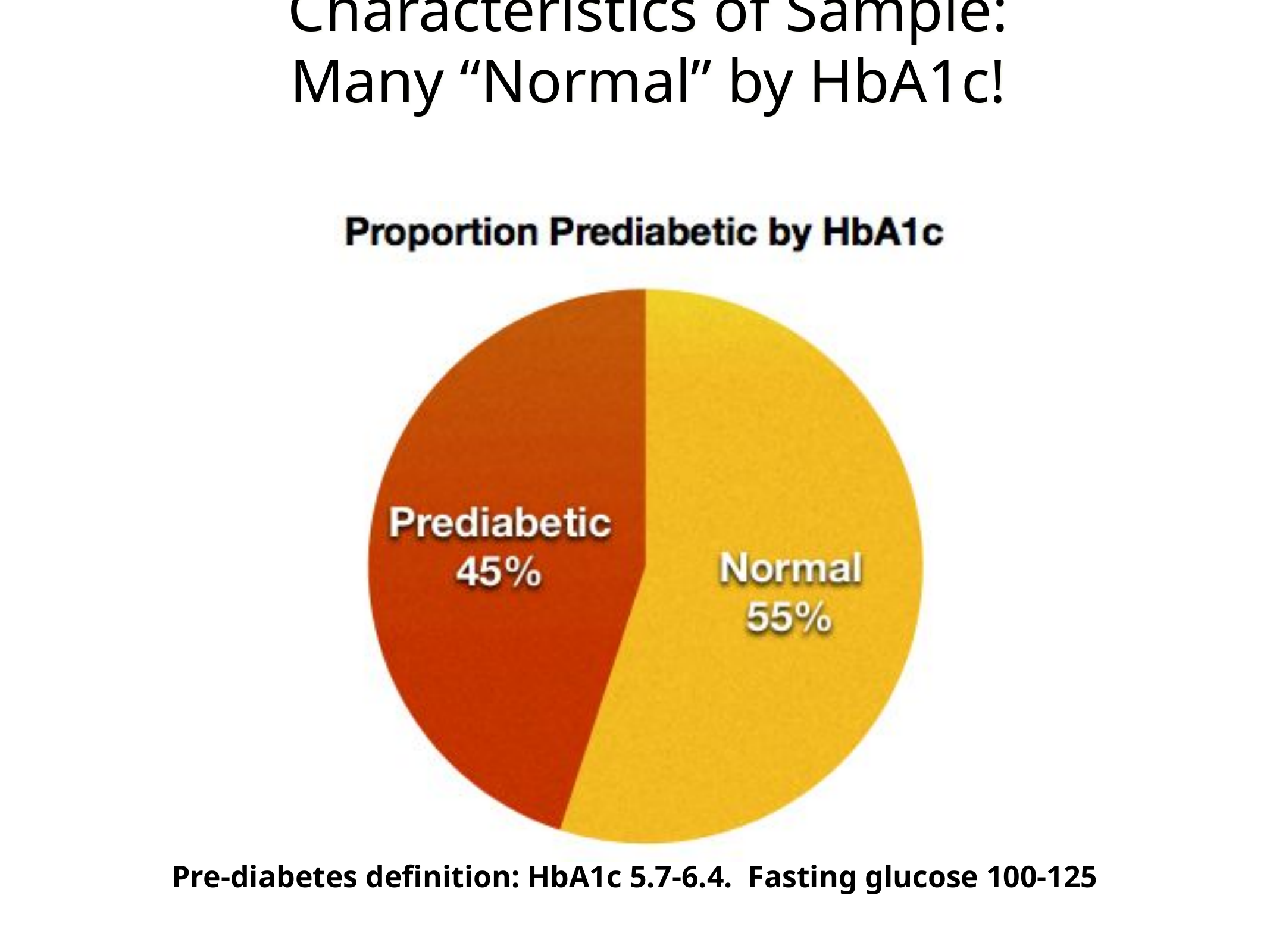

# Characteristics of Sample: Many “Normal” by HbA1c!
Pre-diabetes definition: HbA1c 5.7-6.4. Fasting glucose 100-125

## Slide 12
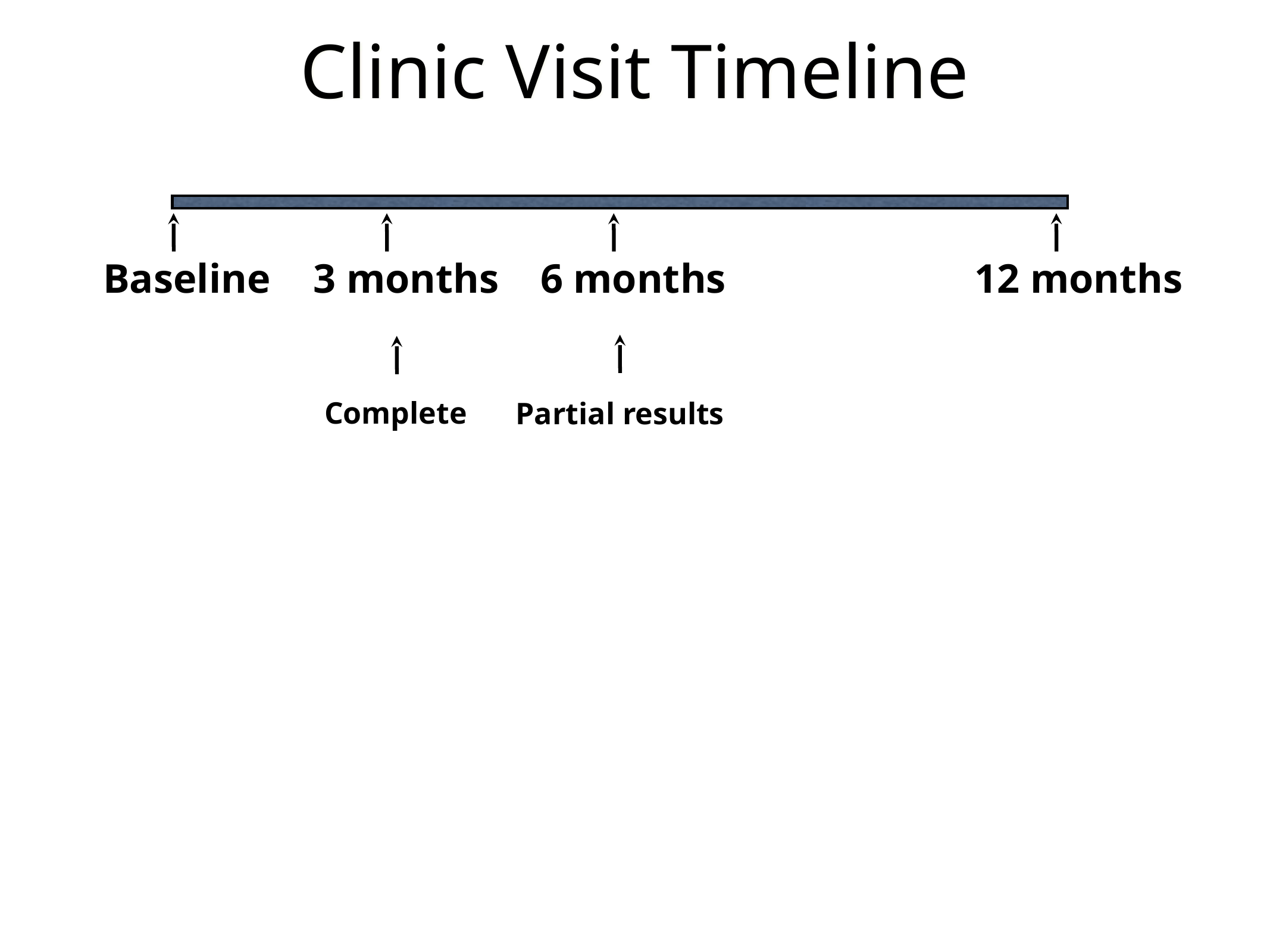

# Clinic Visit Timeline
Baseline
3 months
6 months
12 months
Complete
Partial results

## Slide 13
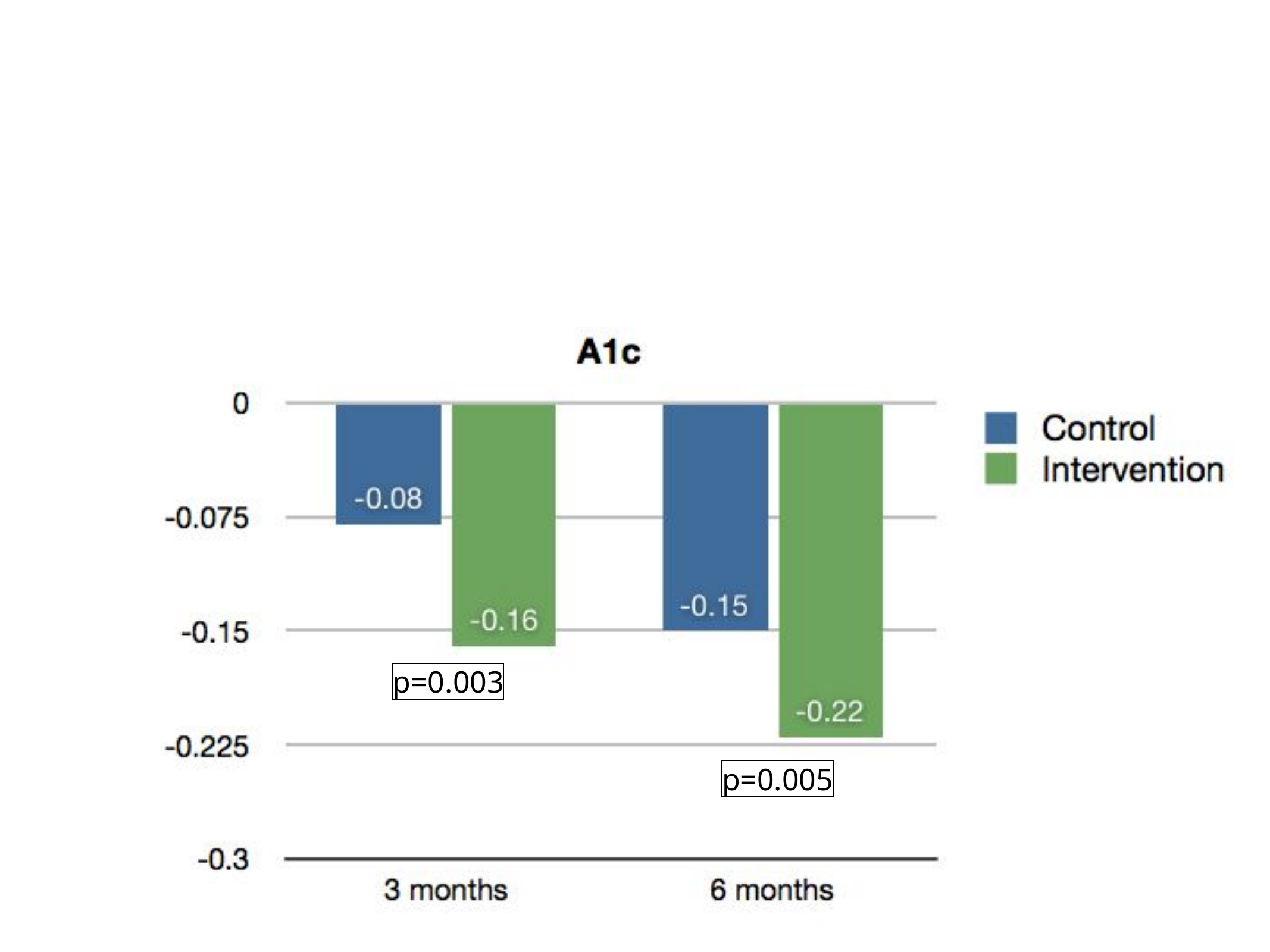

# Alive-PD Treatment Effect: HbA1c
p=0.003
p=0.005

## Slide 14
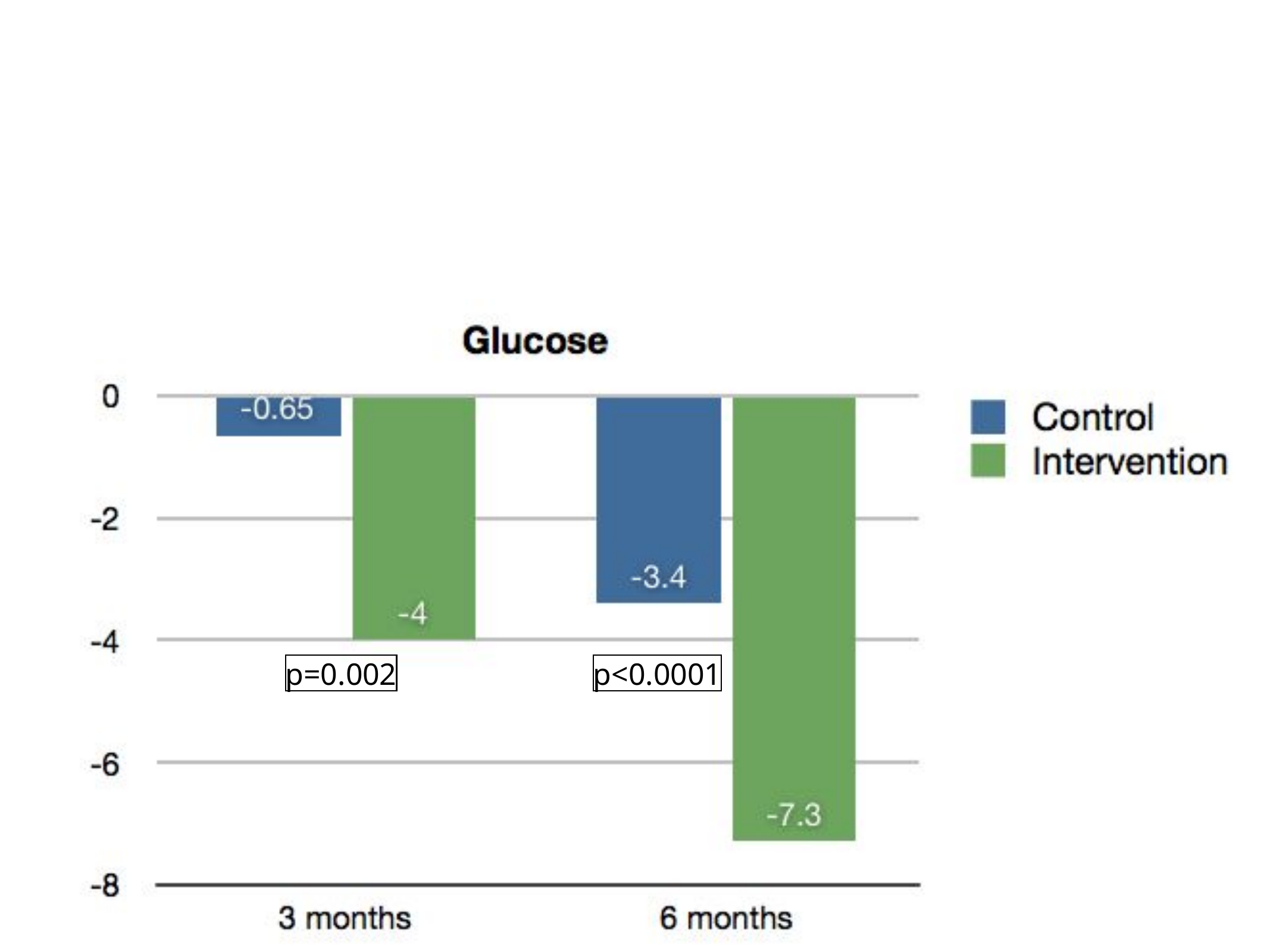

# Alive-PD Treatment Effect: Fasting Glucose
p=0.002
p<0.0001

## Slide 15
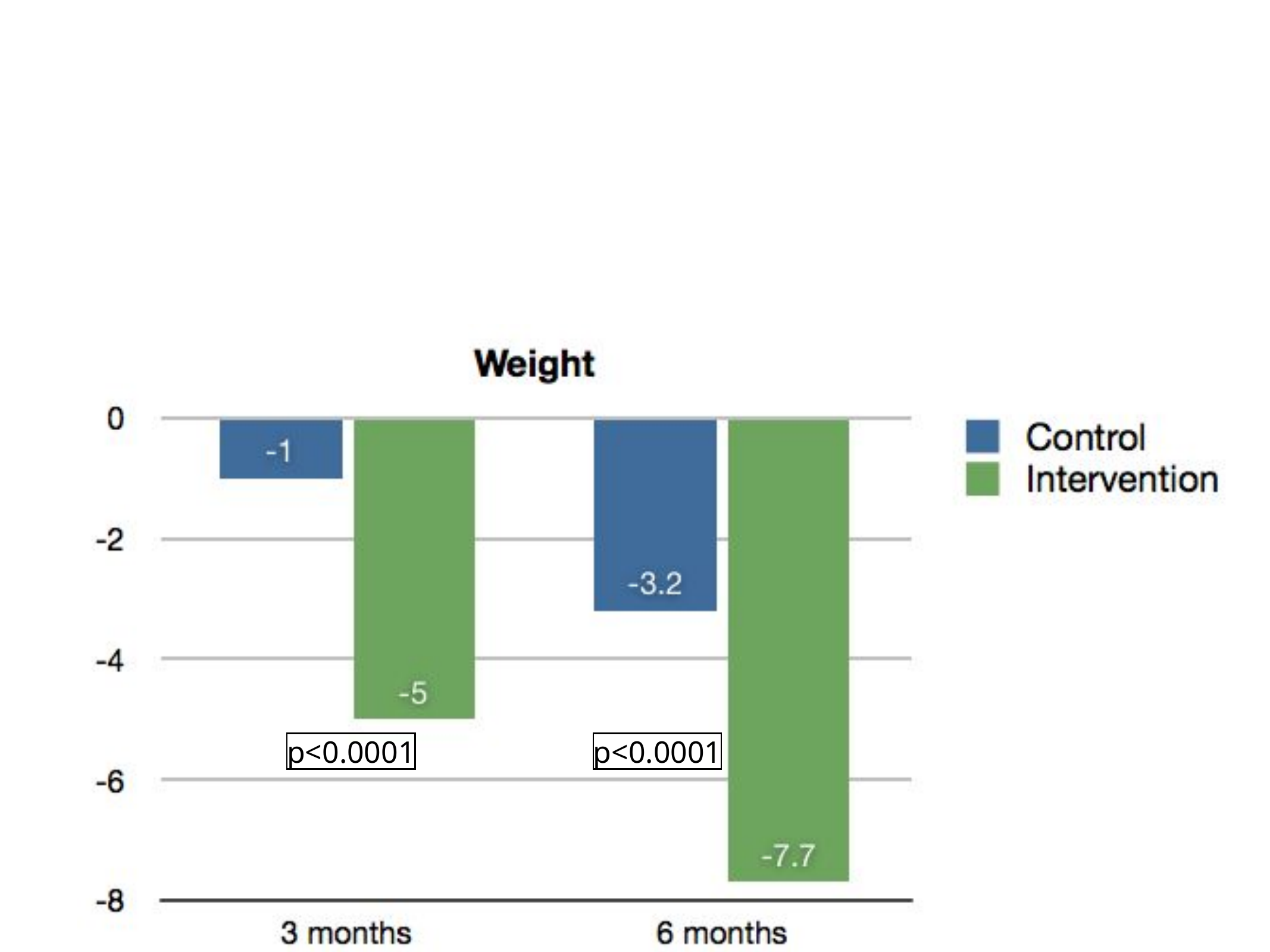

# Alive-PD Treatment Effect: Weight
p<0.0001
p<0.0001

## Slide 16
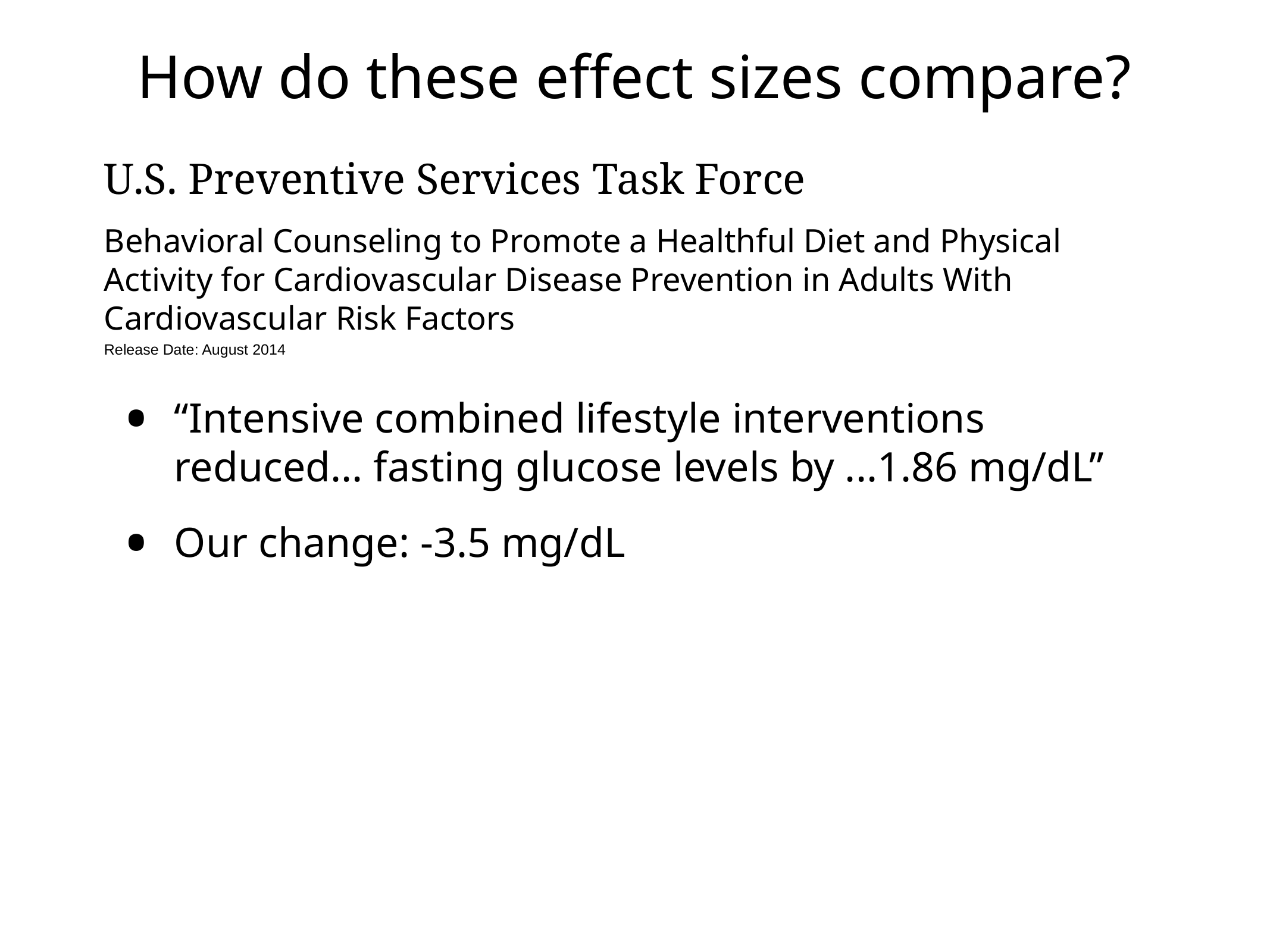

# How do these effect sizes compare?
U.S. Preventive Services Task Force
Behavioral Counseling to Promote a Healthful Diet and Physical Activity for Cardiovascular Disease Prevention in Adults With Cardiovascular Risk Factors
Release Date: August 2014
“Intensive combined lifestyle interventions reduced... fasting glucose levels by ...1.86 mg/dL”
Our change: -3.5 mg/dL

## Slide 17
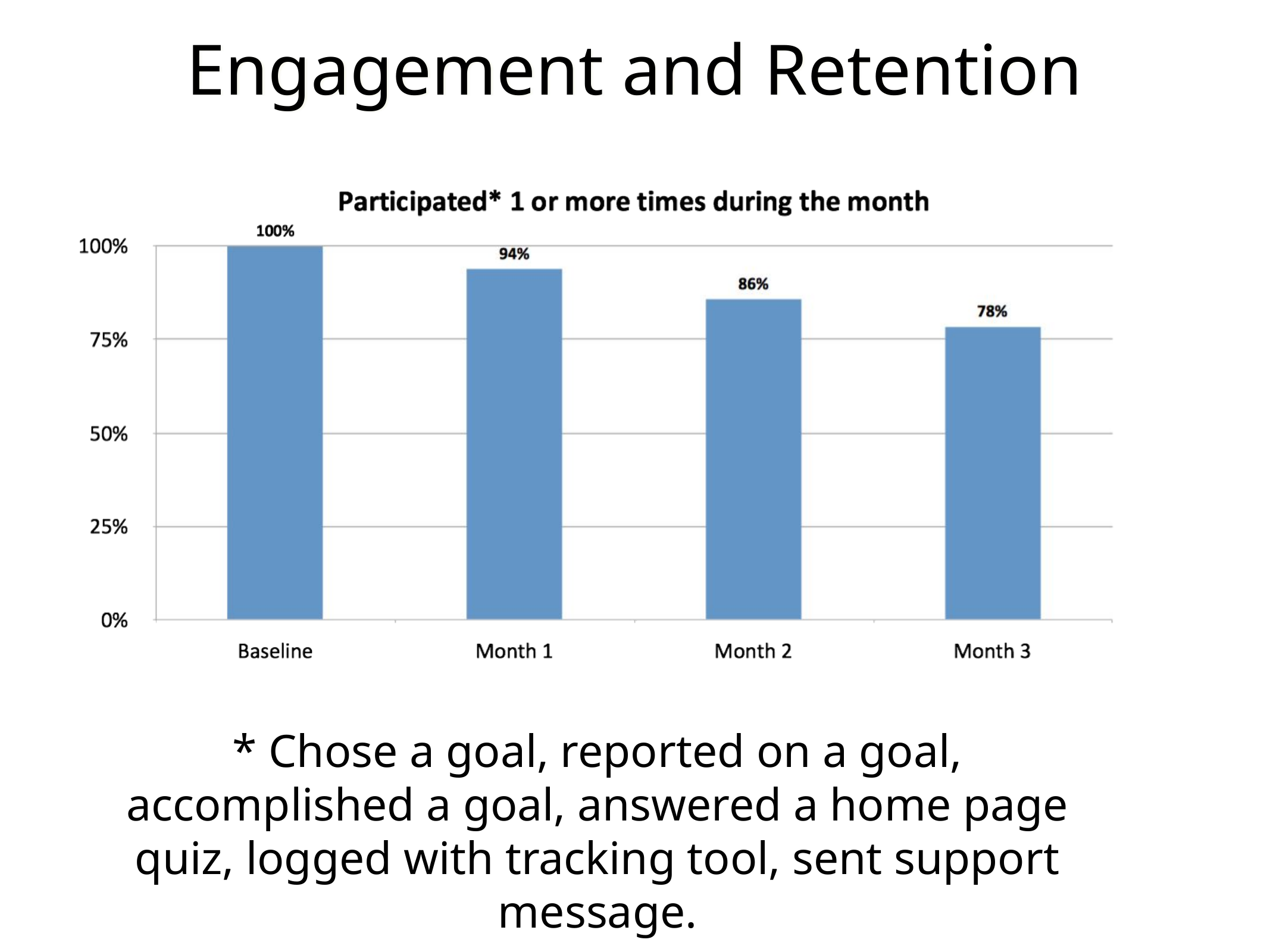

Engagement and Retention
* Chose a goal, reported on a goal, accomplished a goal, answered a home page quiz, logged with tracking tool, sent support message.

## Slide 18
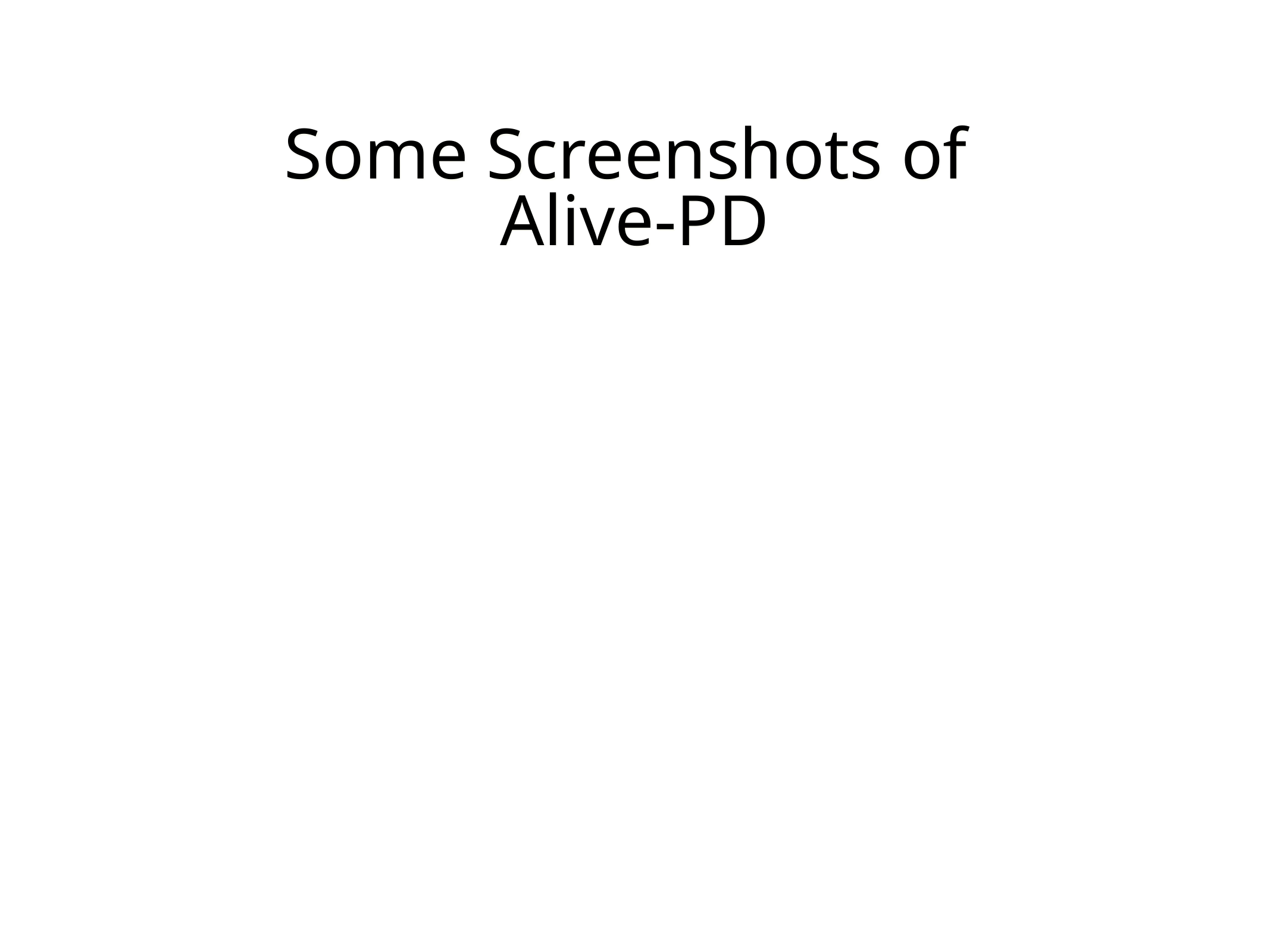

Some Screenshots of
Alive-PD

## Slide 19
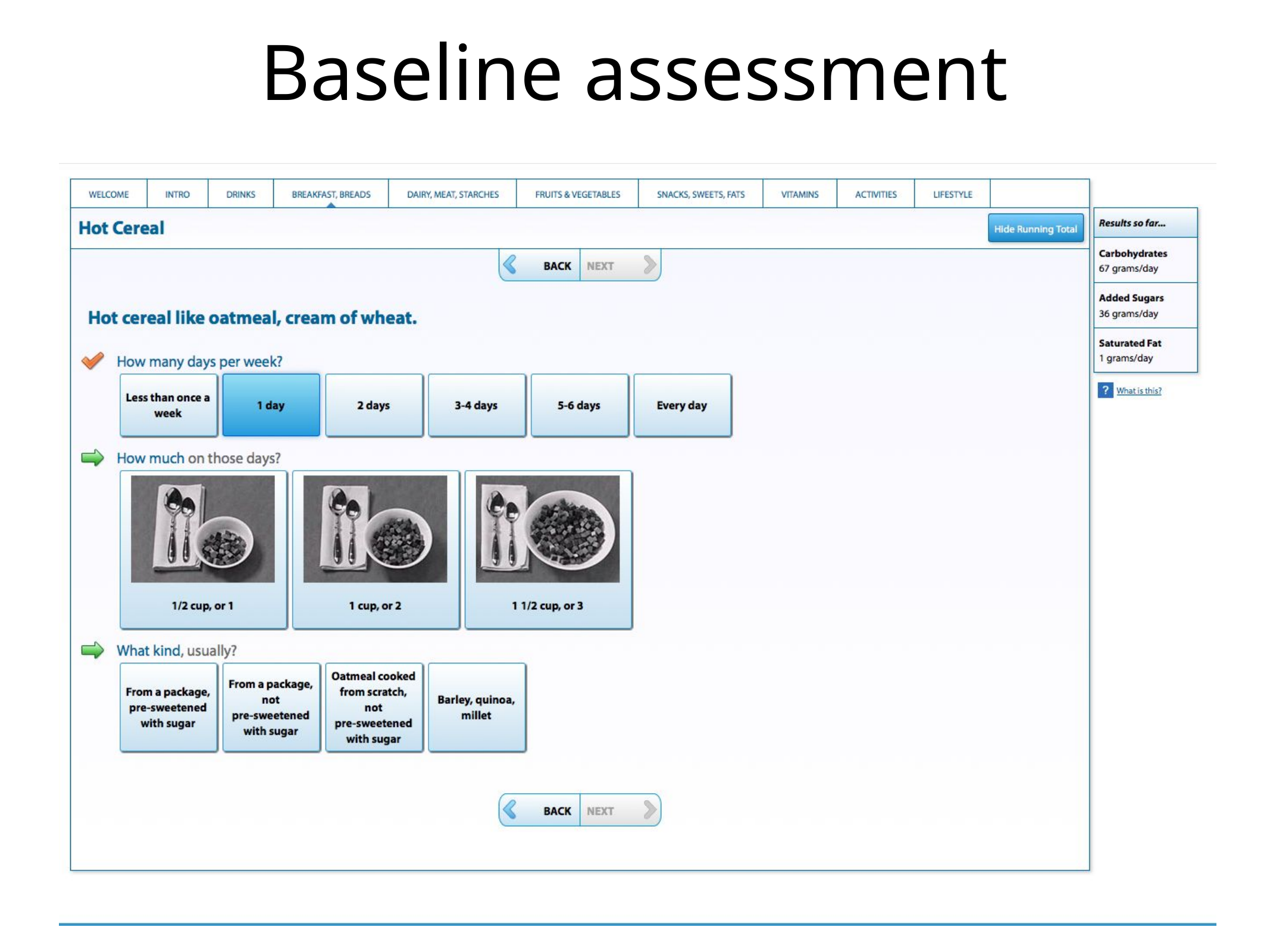

# Baseline assessment

## Slide 20
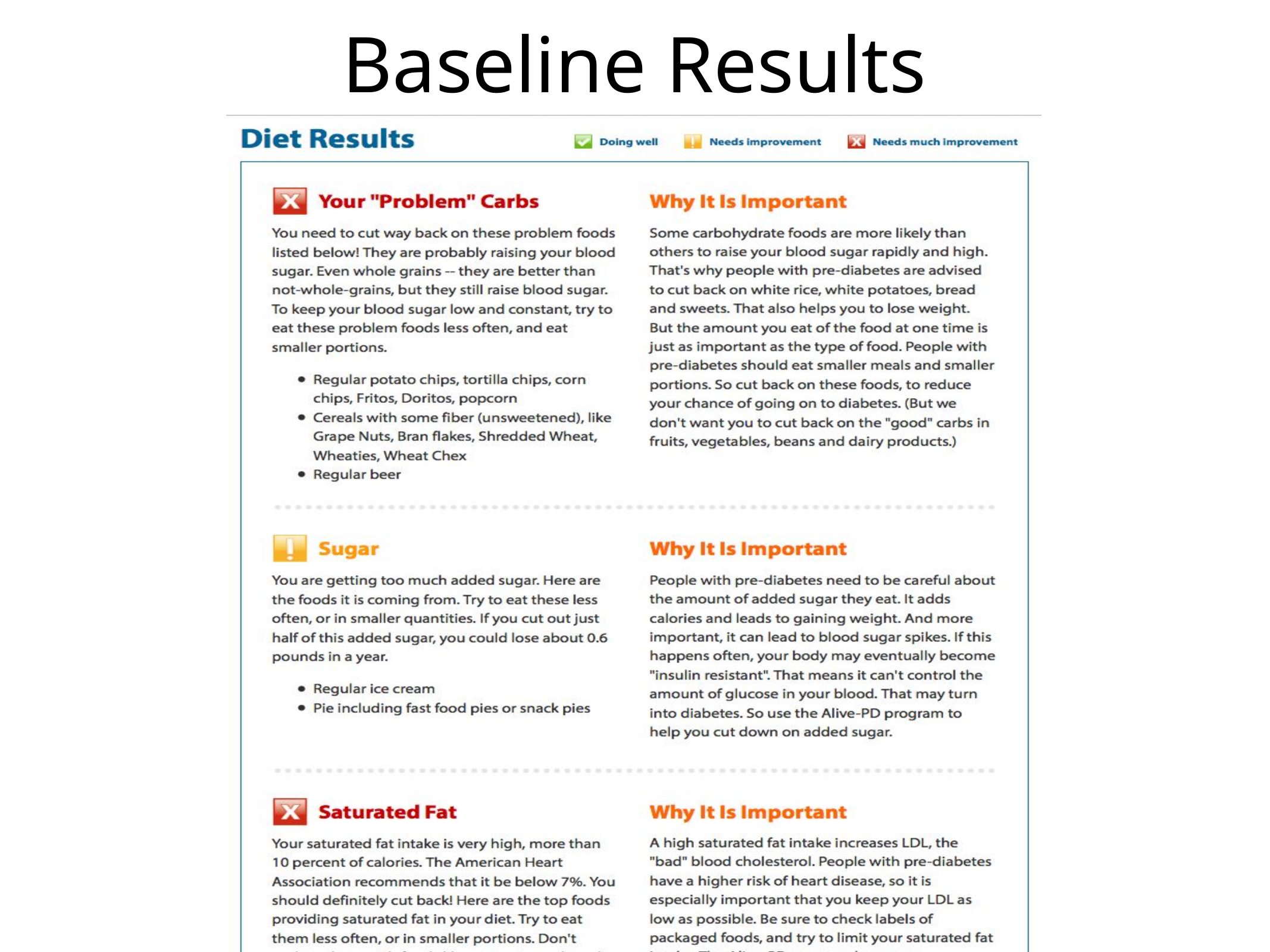

# Baseline Results

## Slide 21
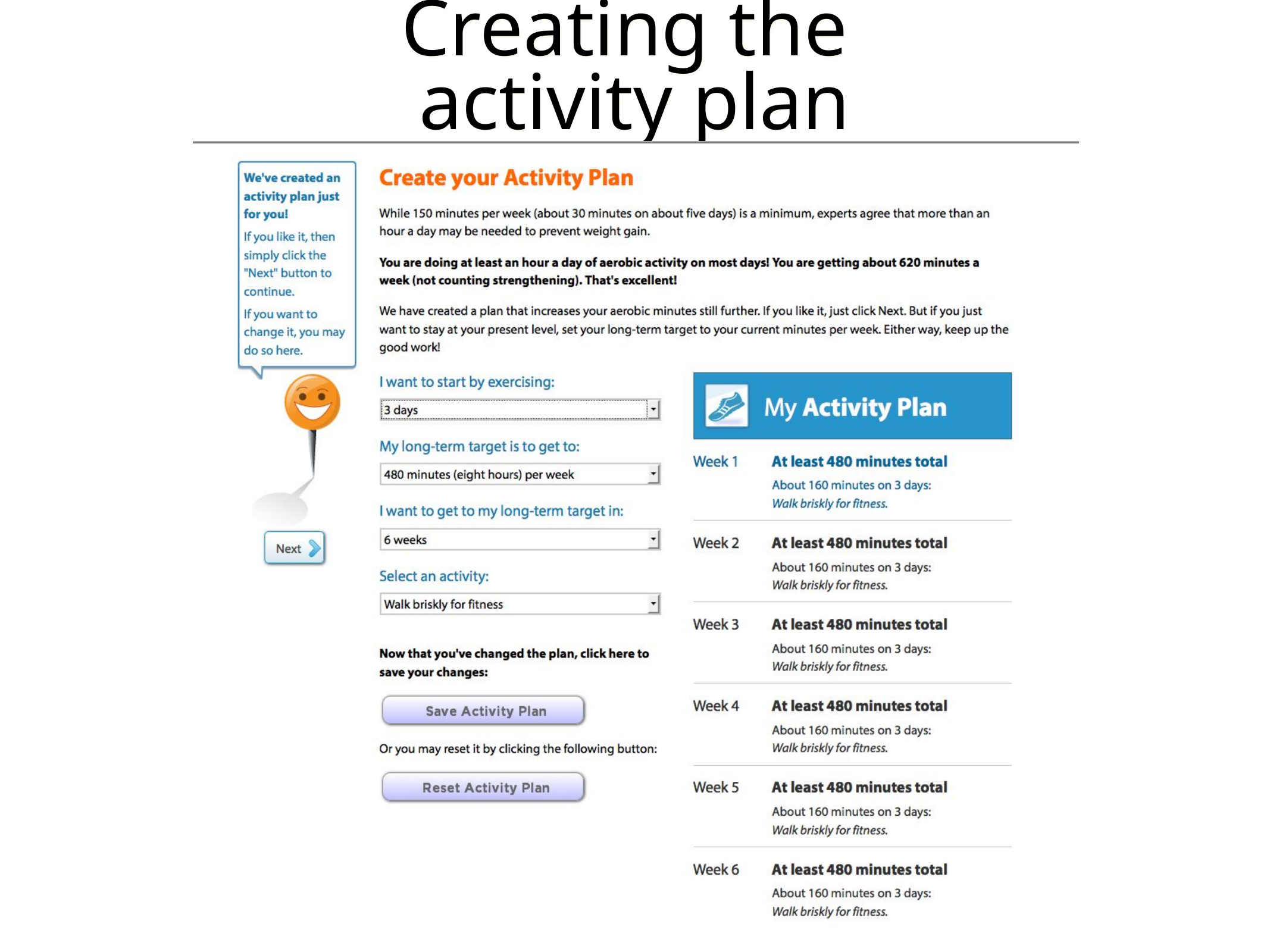

# Creating the activity plan

## Slide 22
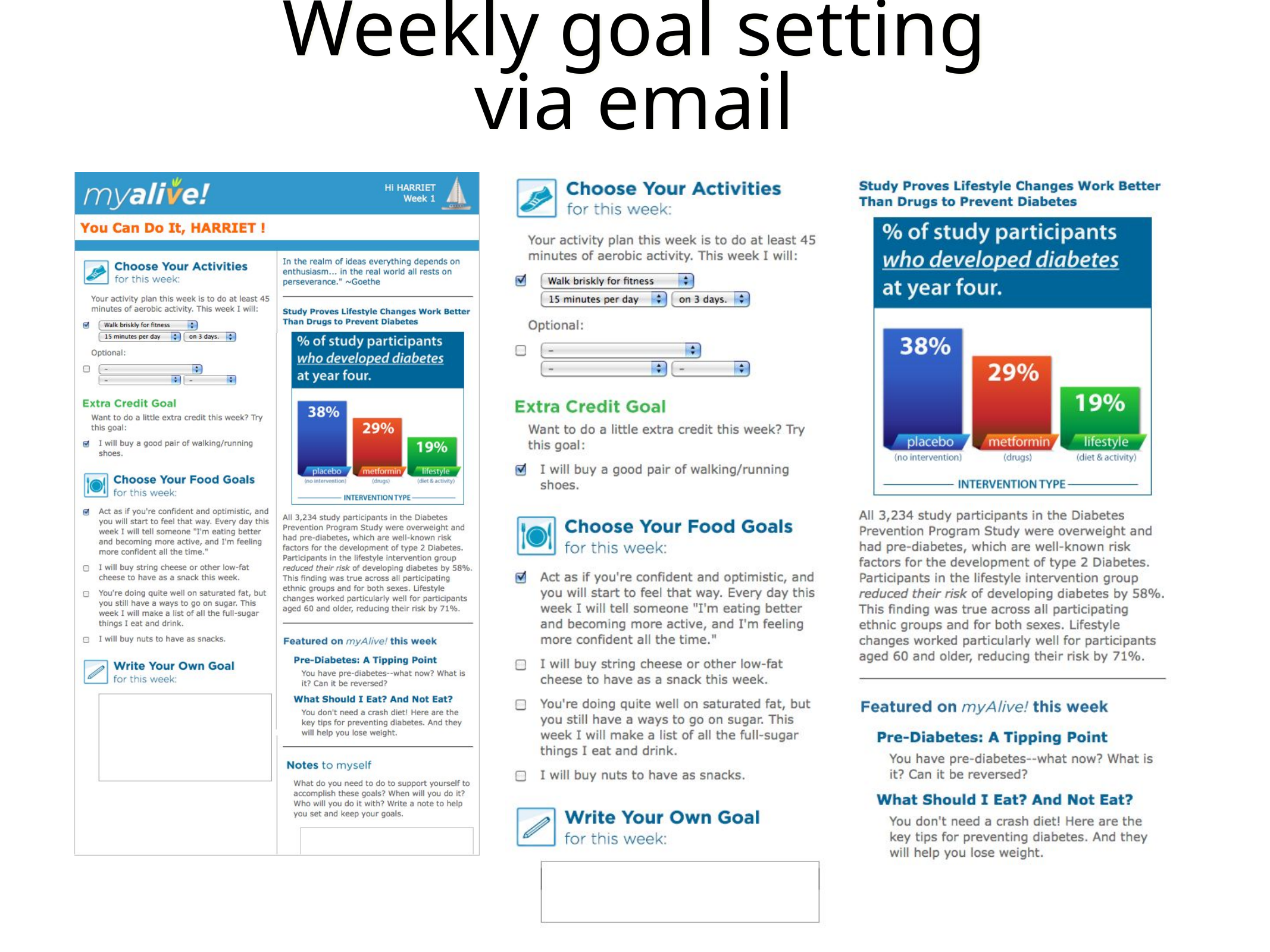

# Weekly goal setting via email

## Slide 23
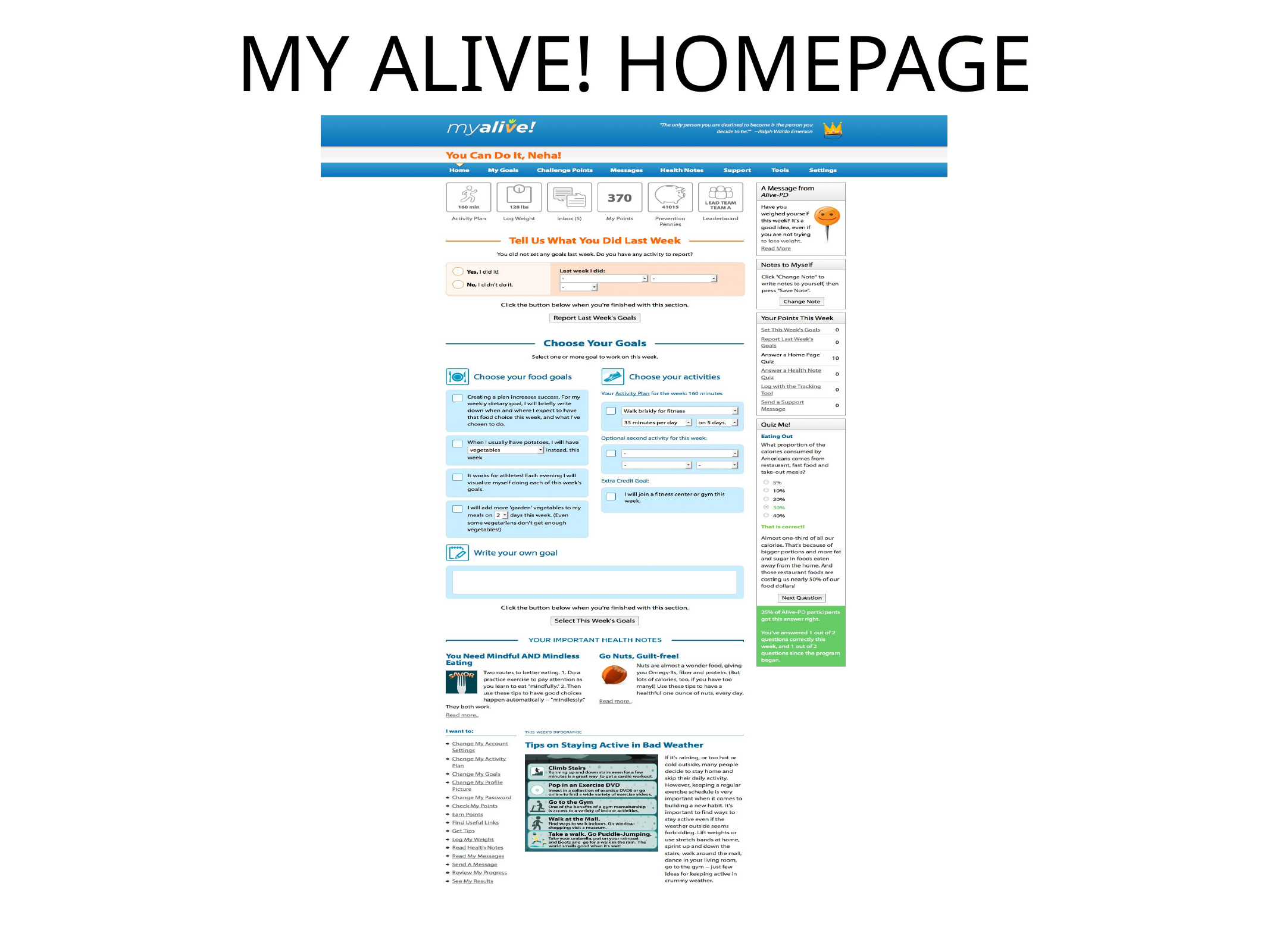

# MY ALIVE! HOMEPAGE

## Slide 24
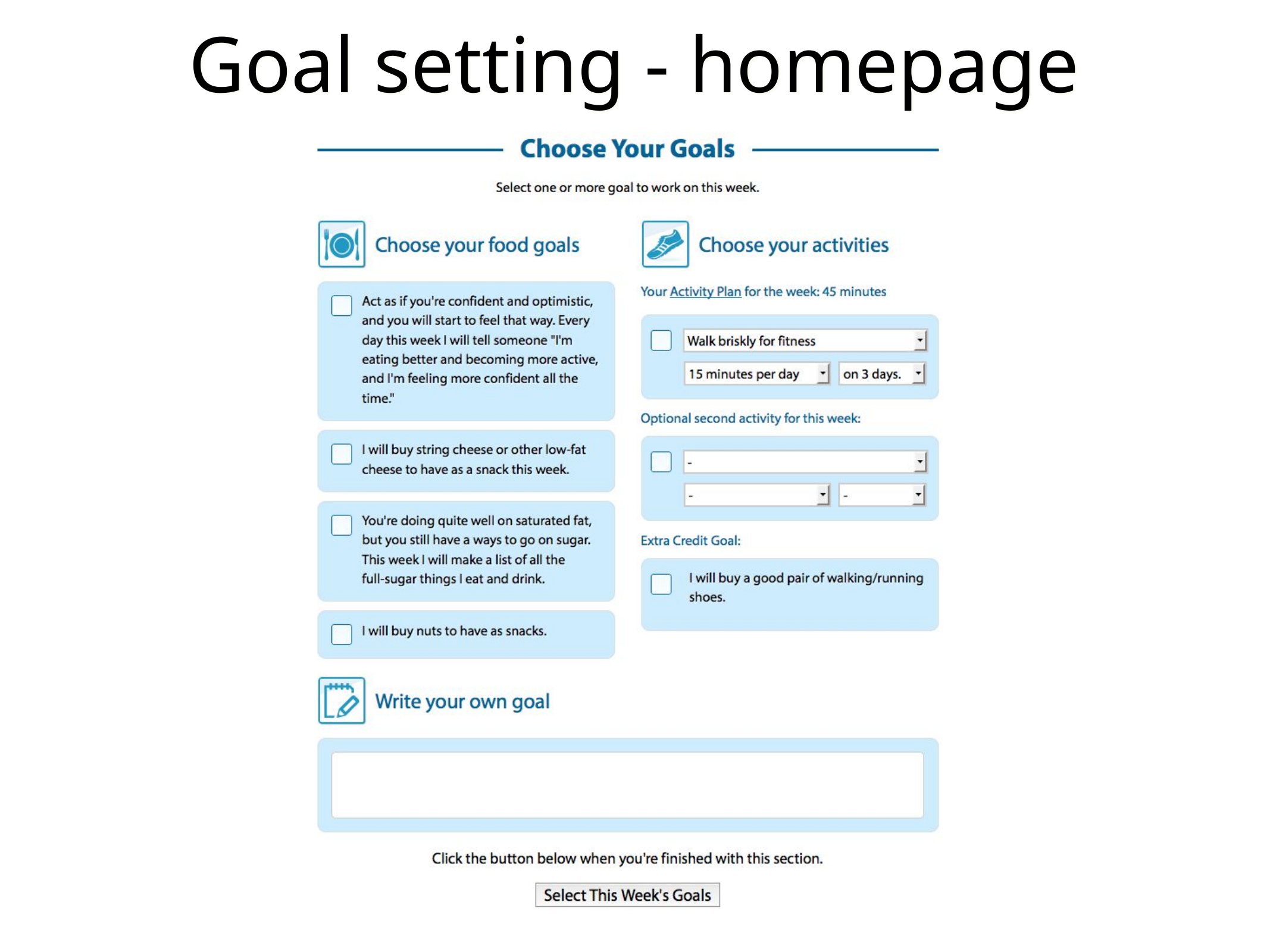

# Goal setting - homepage

## Slide 25
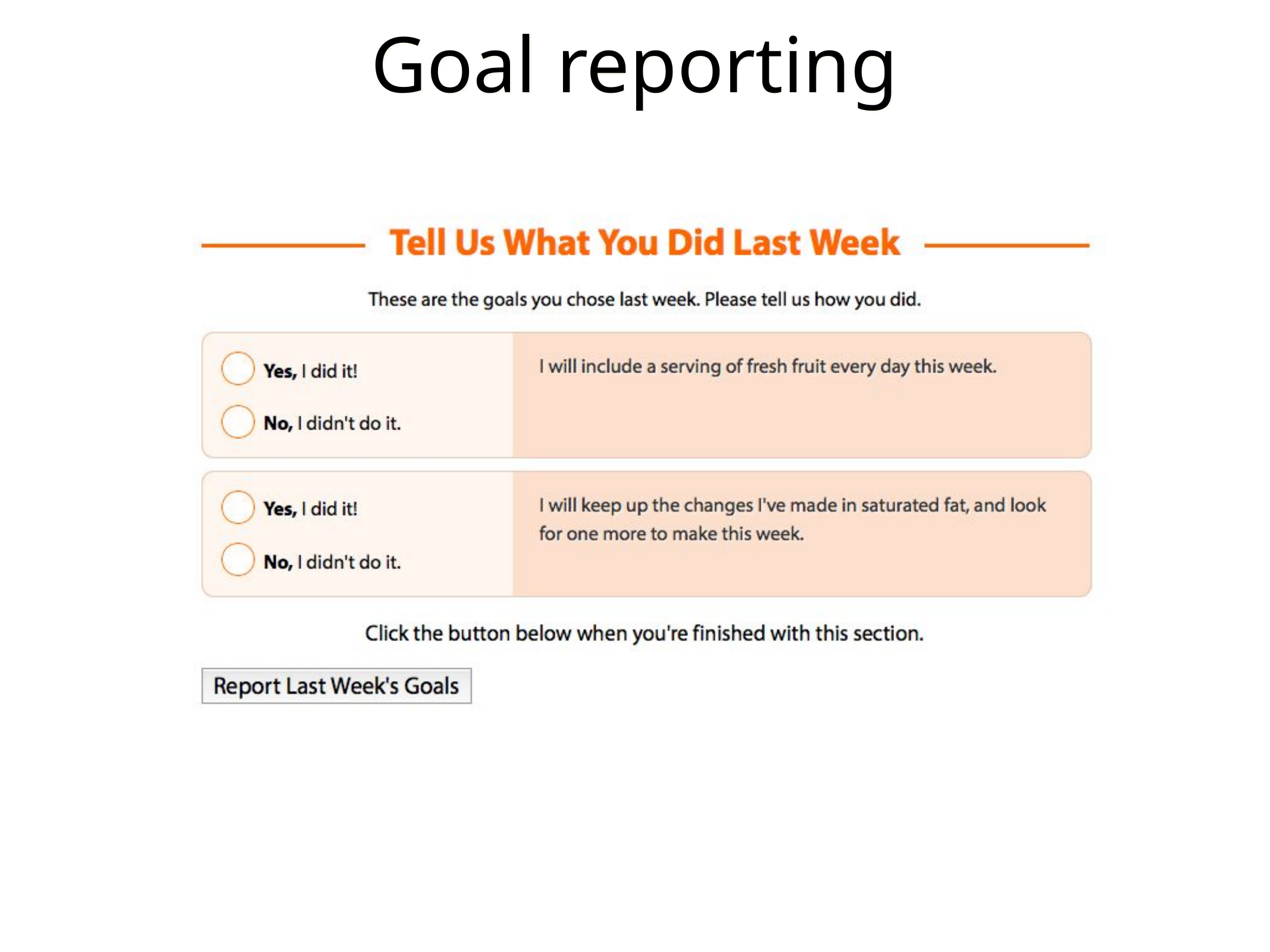

# Goal reporting

## Slide 26
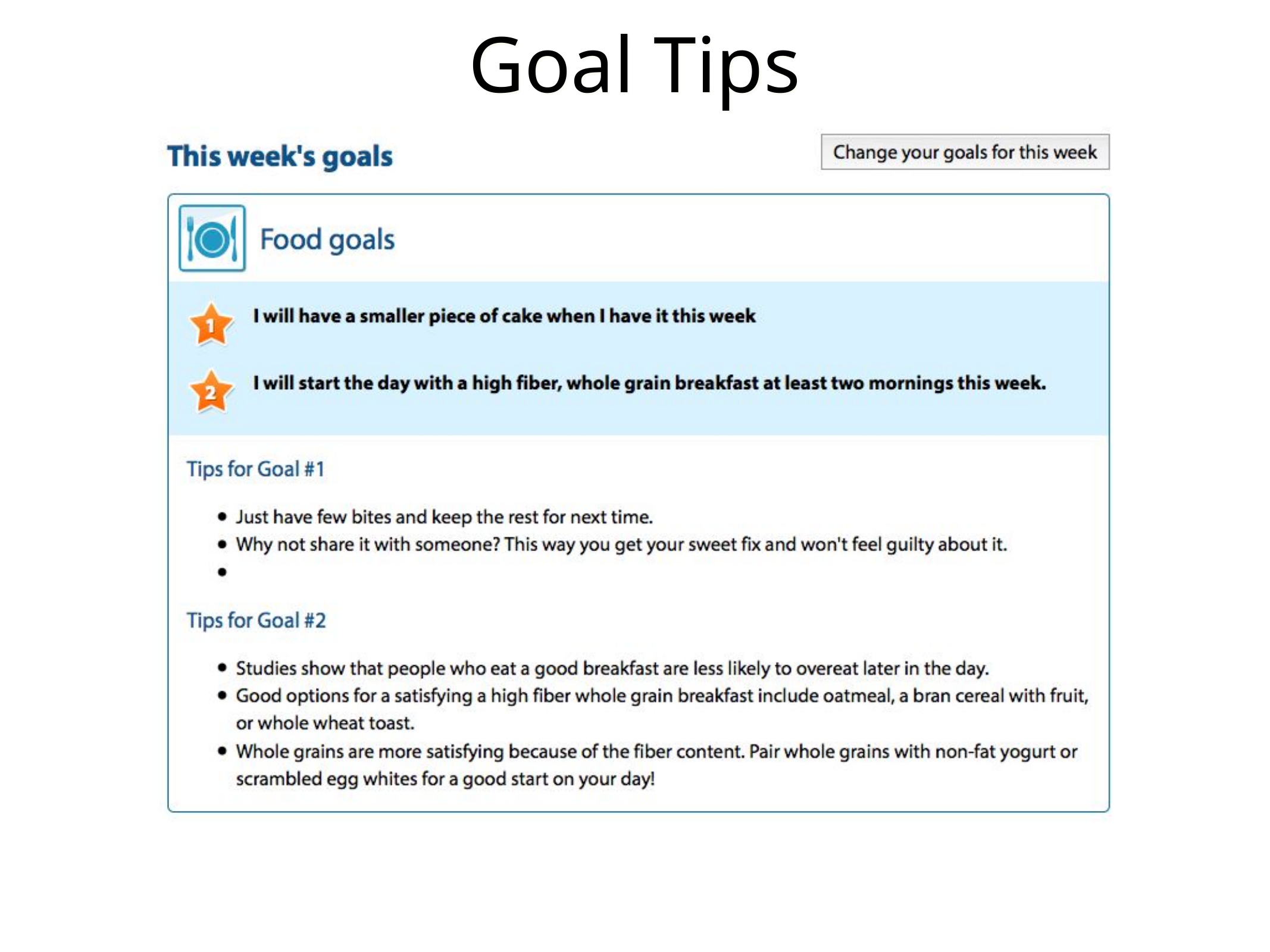

# Goal Tips

## Slide 27
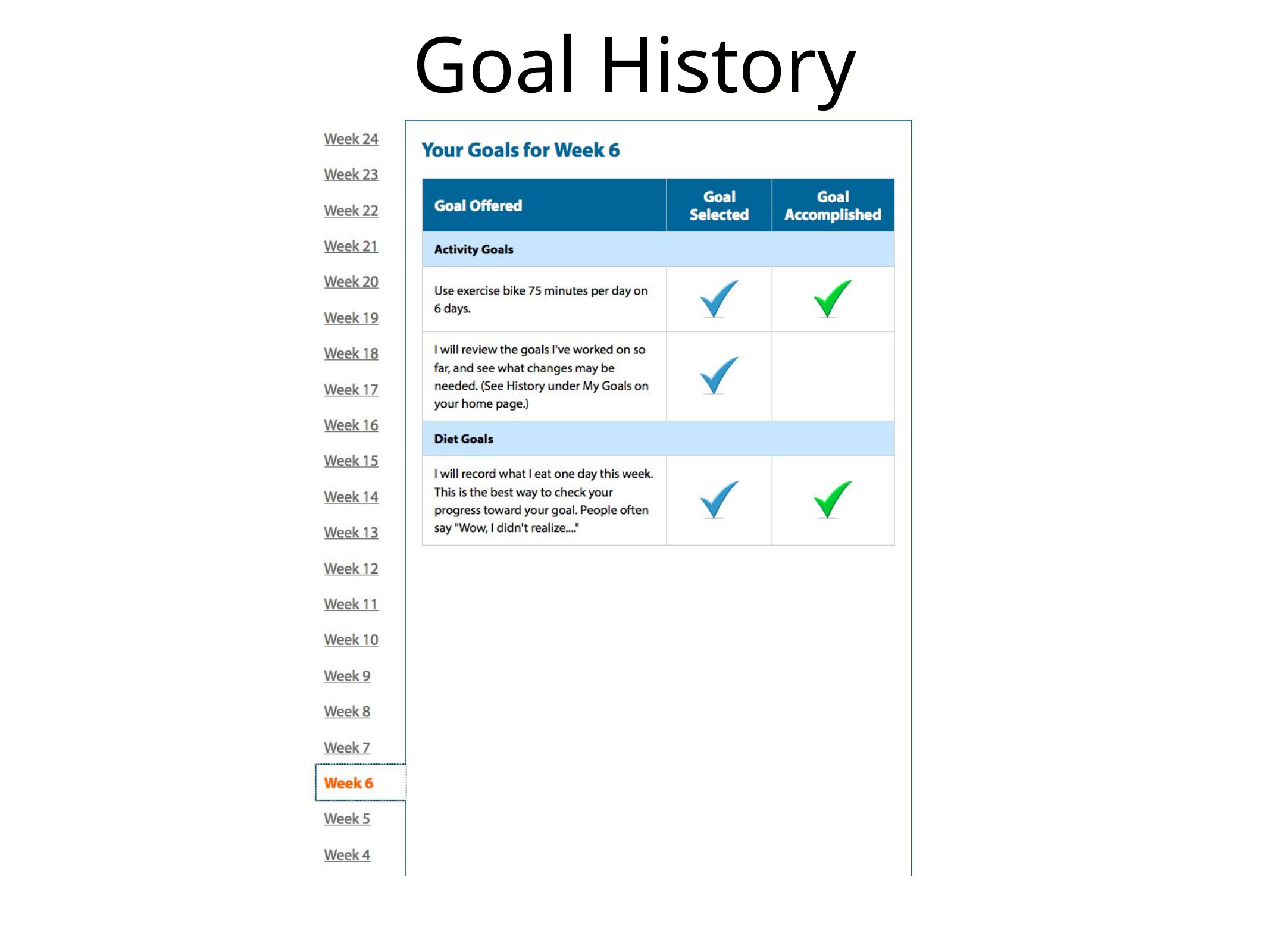

# Goal History

## Slide 28
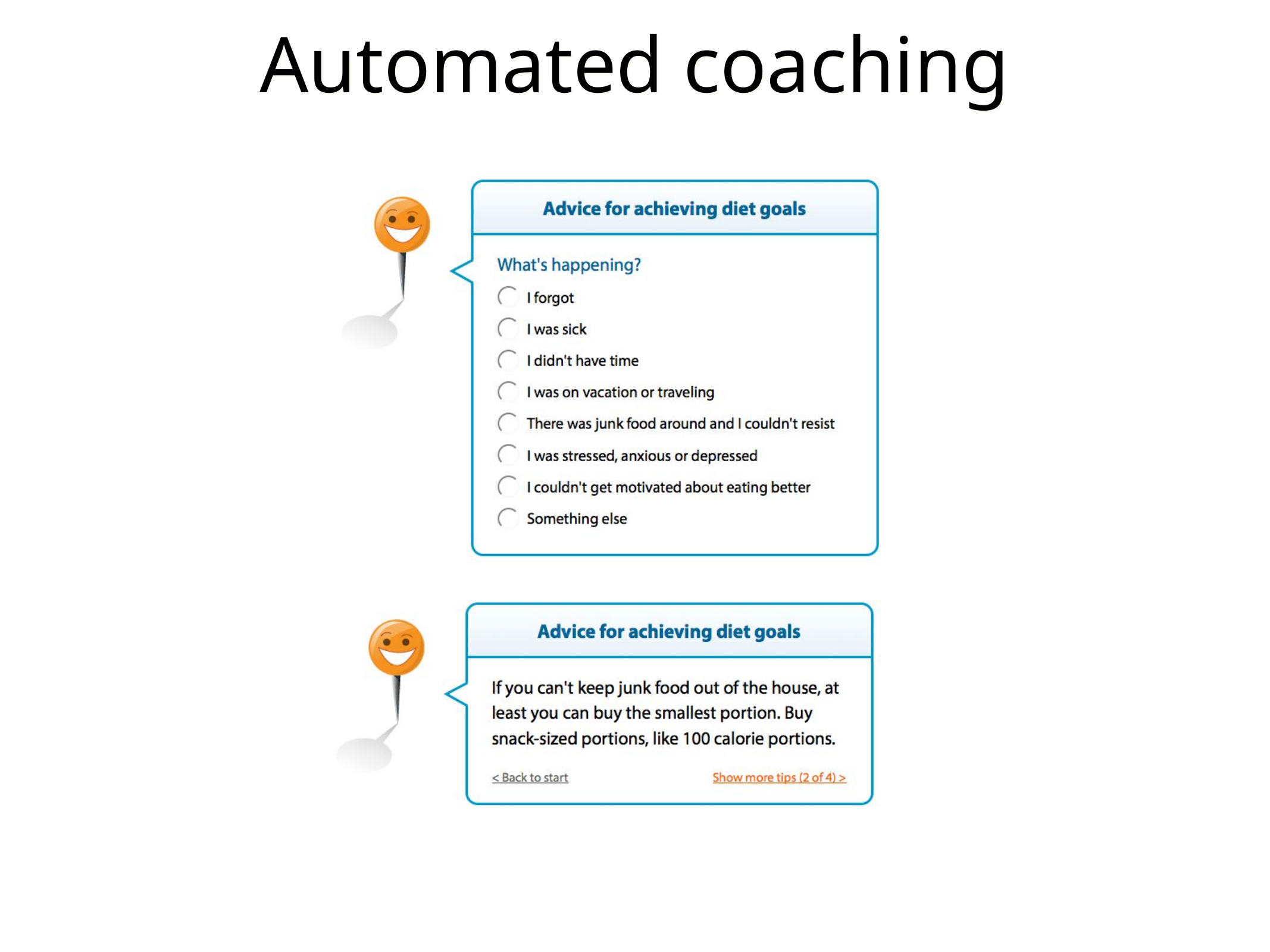

# Automated coaching

## Slide 29
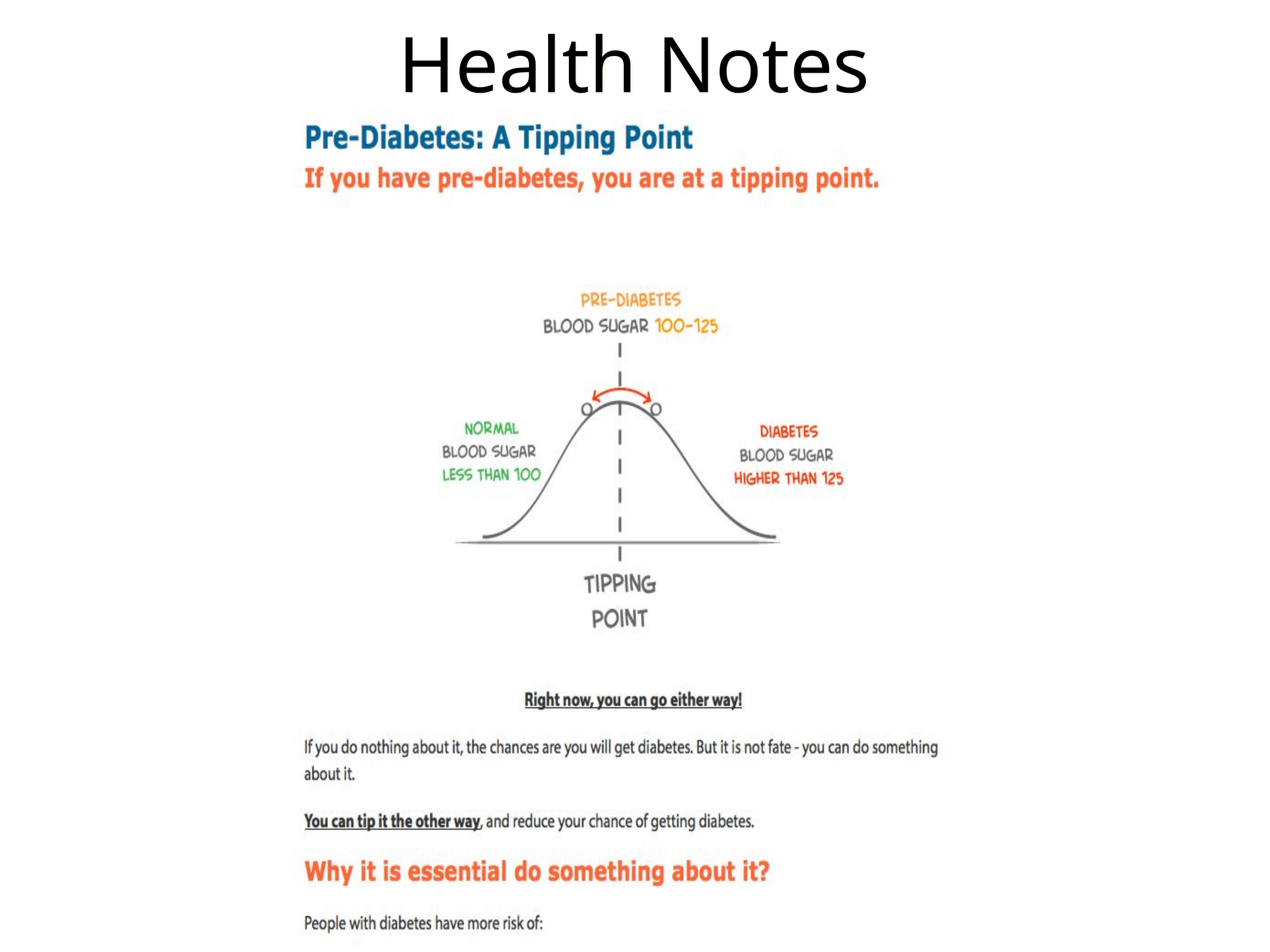

# Health Notes

## Slide 30
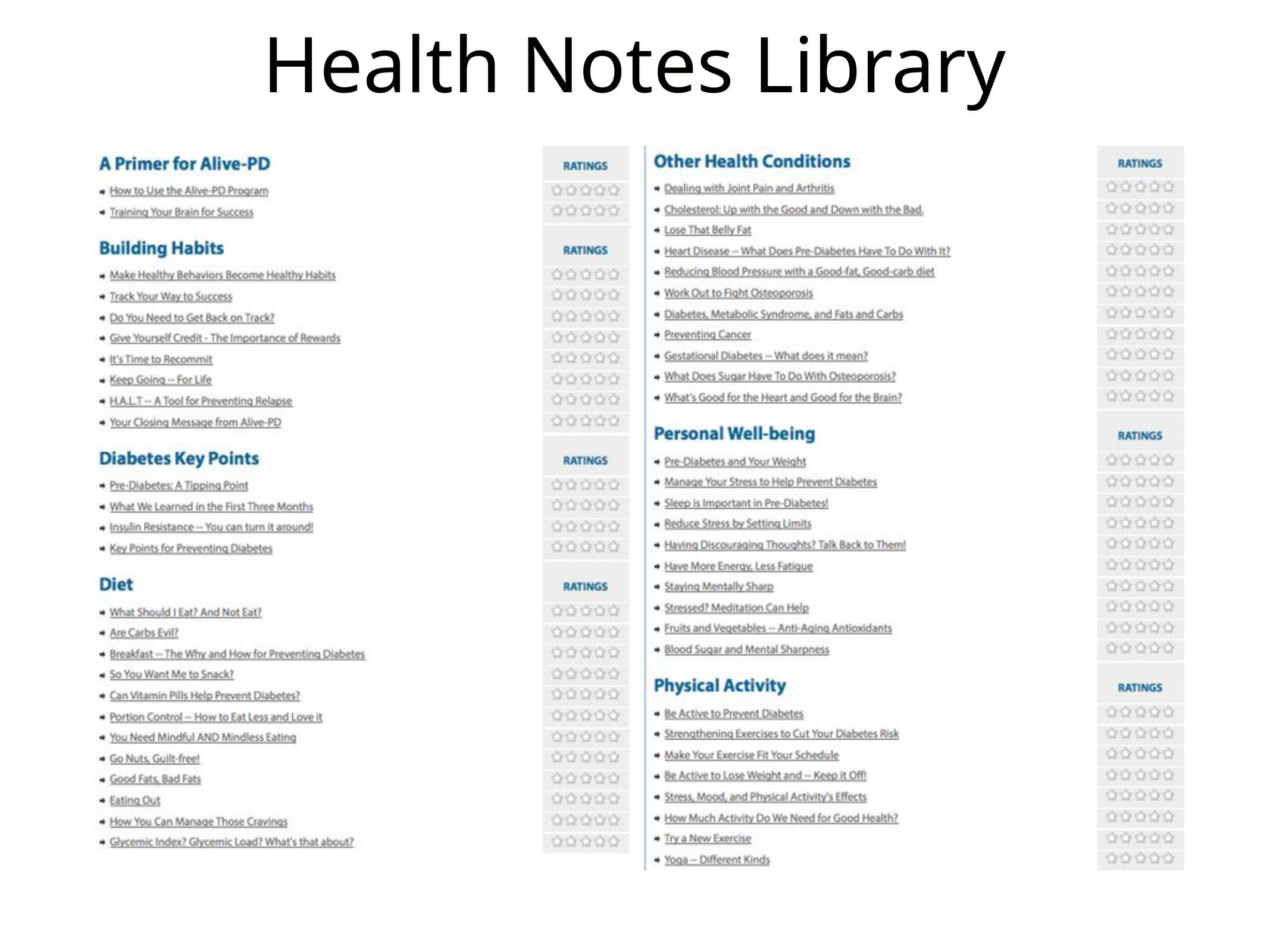

# Health Notes Library

## Slide 31
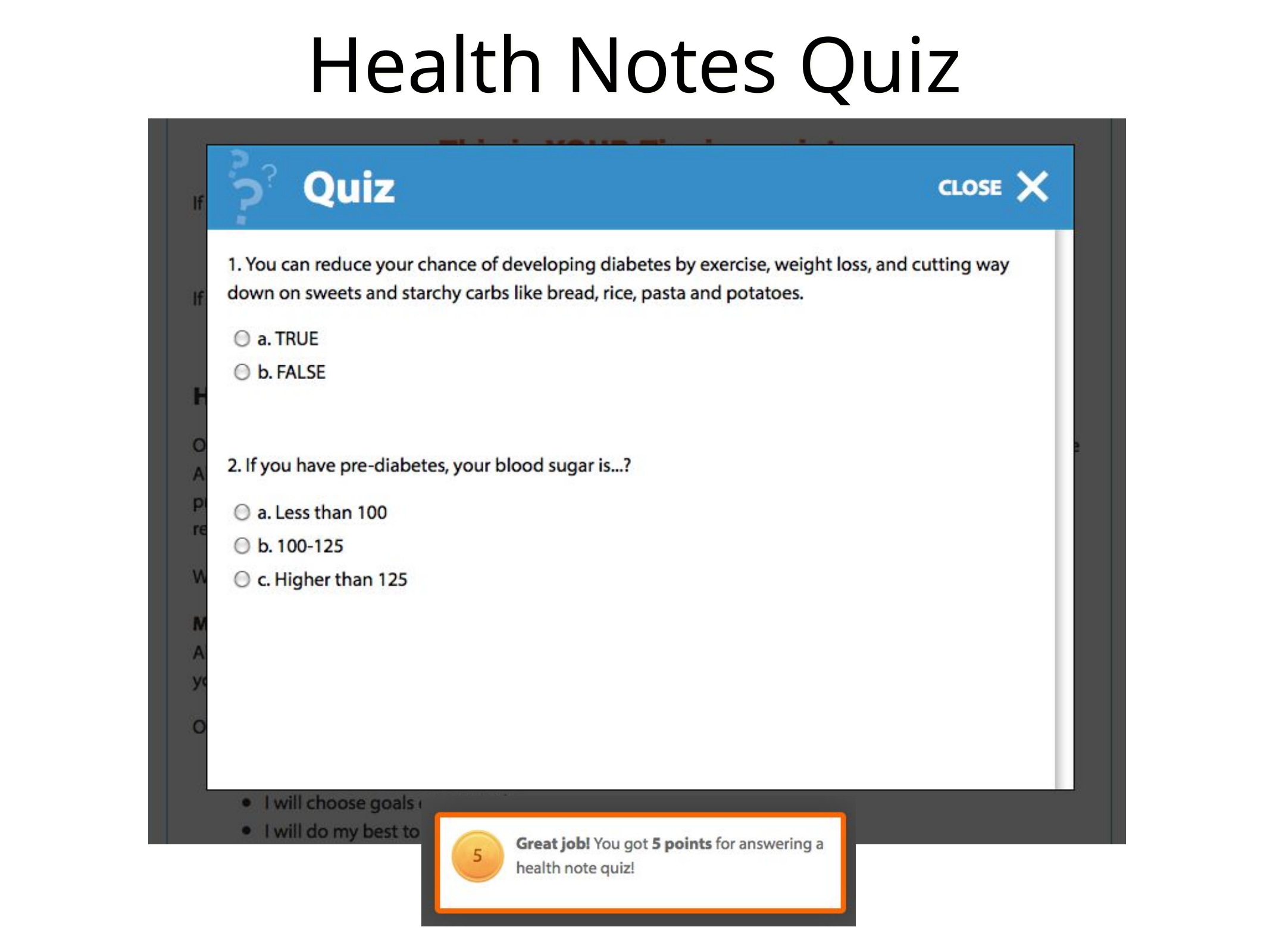

# Health Notes Quiz

## Slide 32
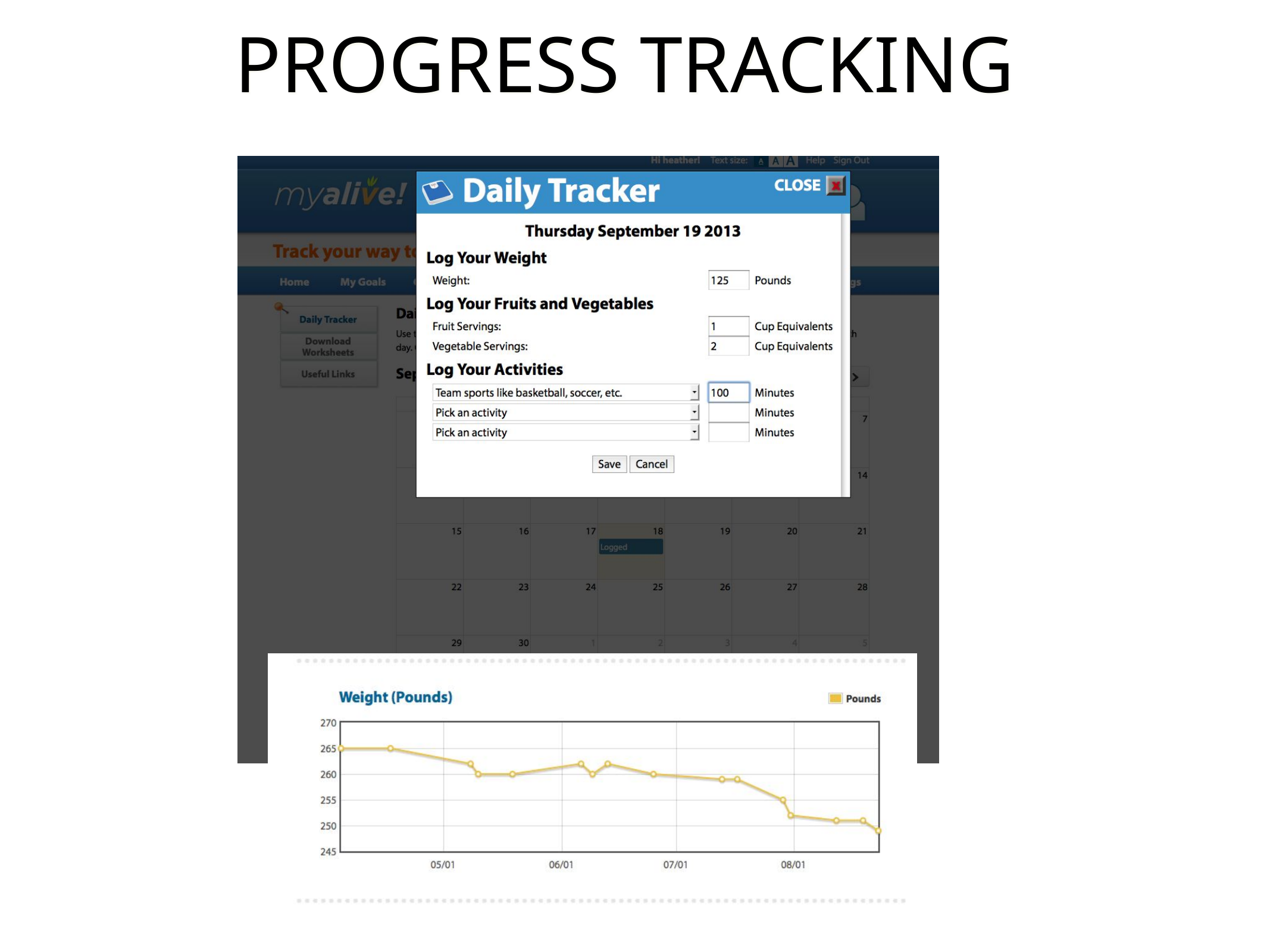

# PROGRESS TRACKING

## Slide 33
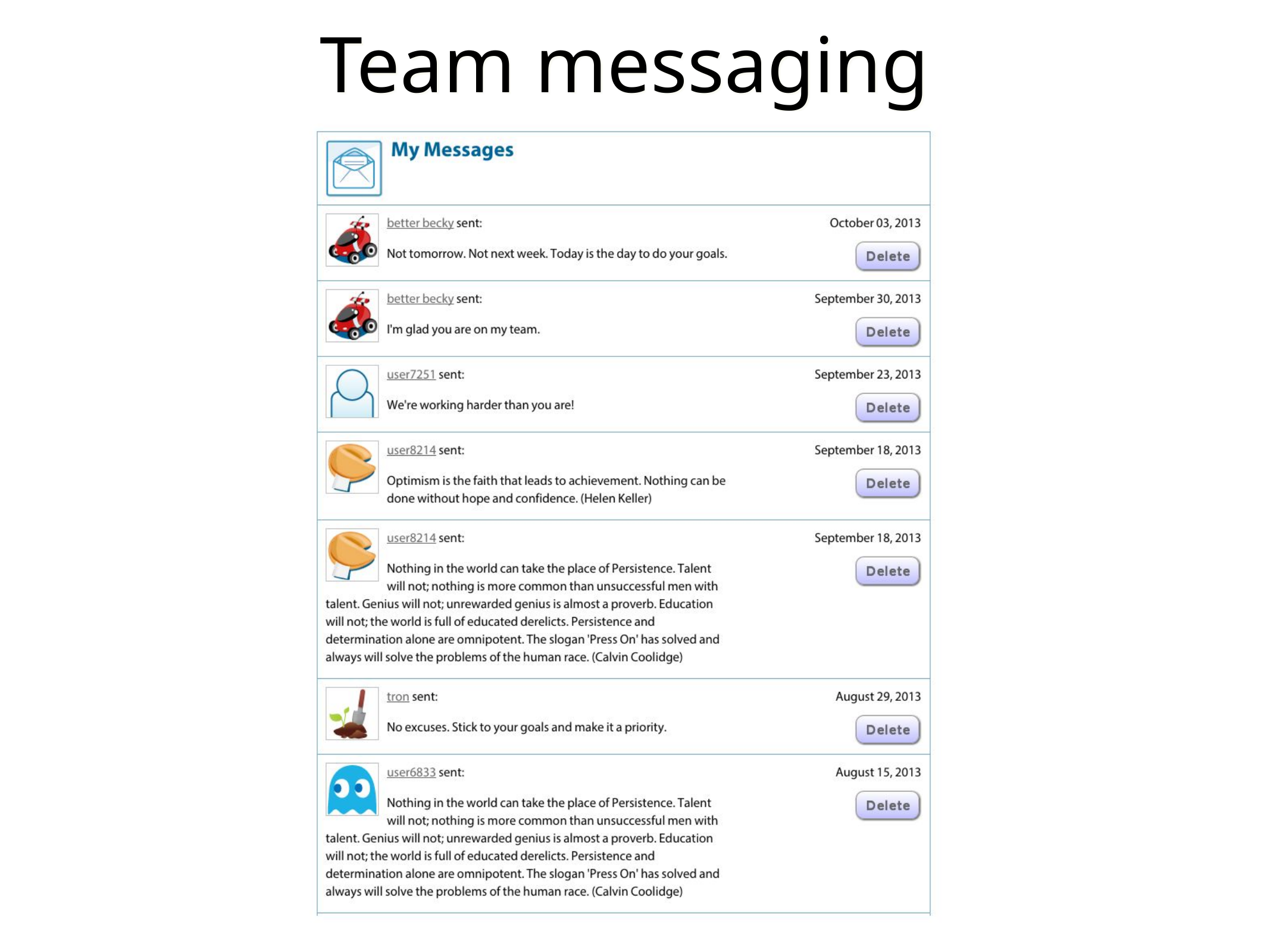

# Team messaging

## Slide 34
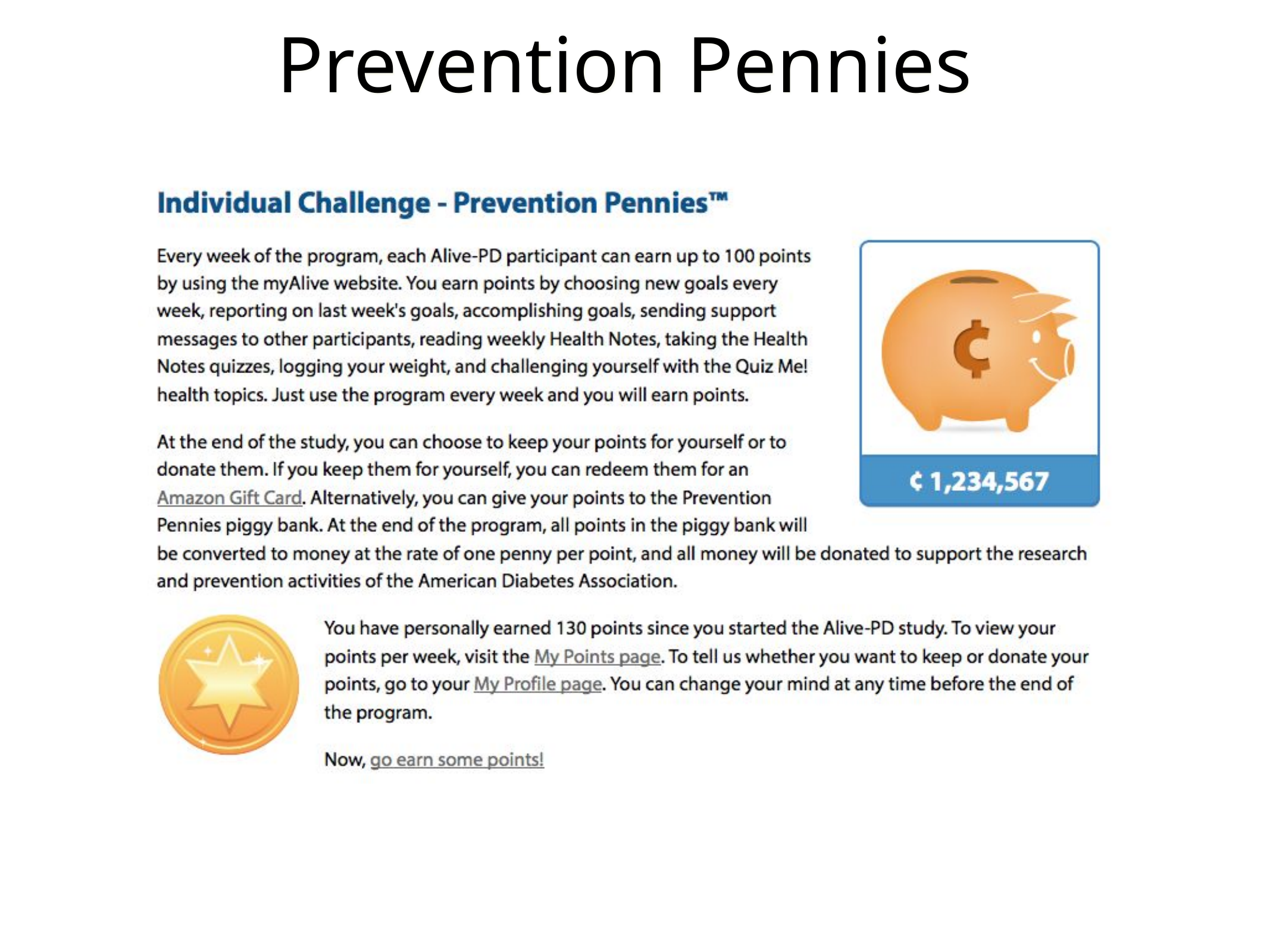

# Prevention Pennies

## Slide 35
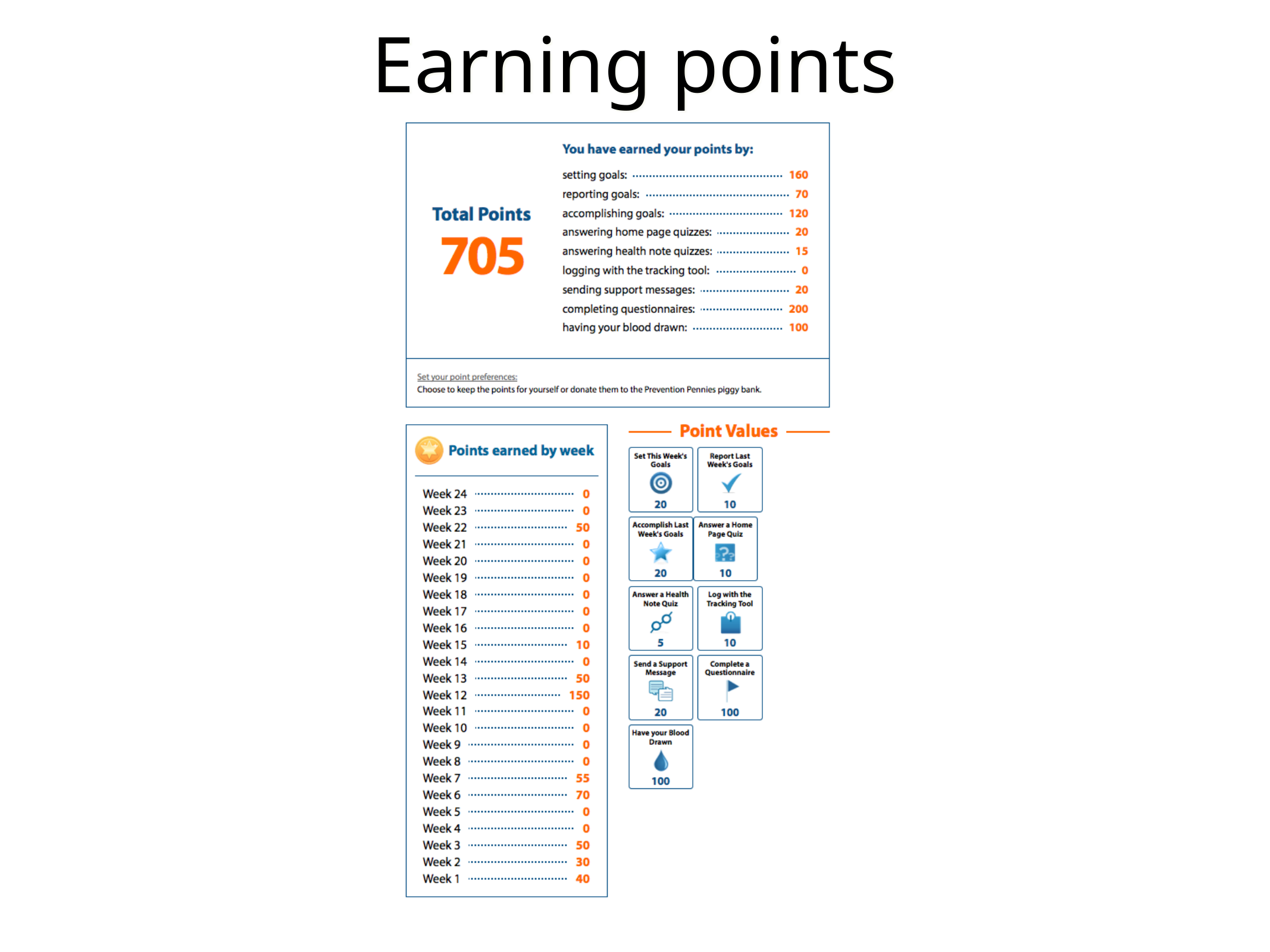

# Earning points

## Slide 36
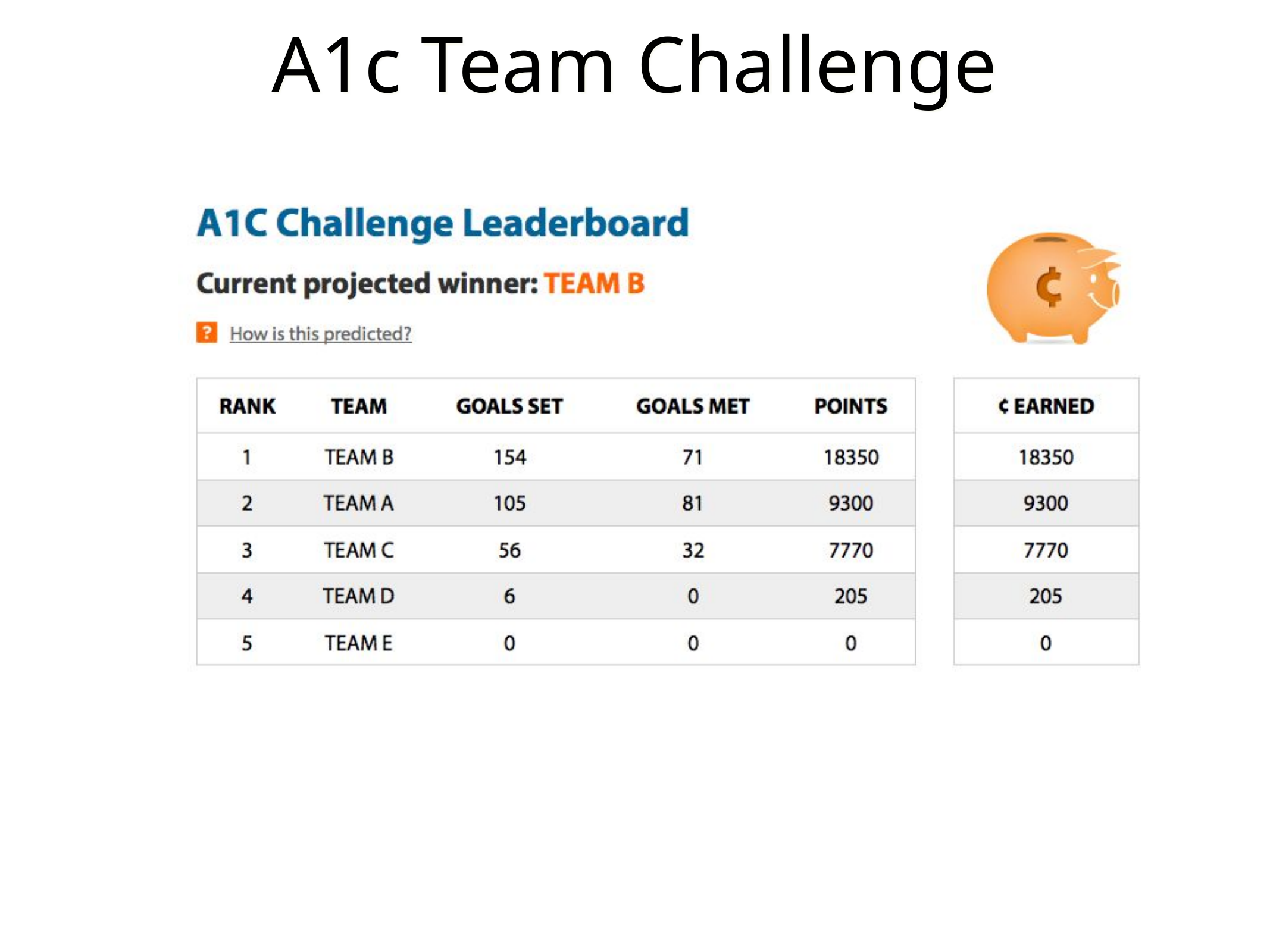

# A1c Team Challenge

## Slide 37
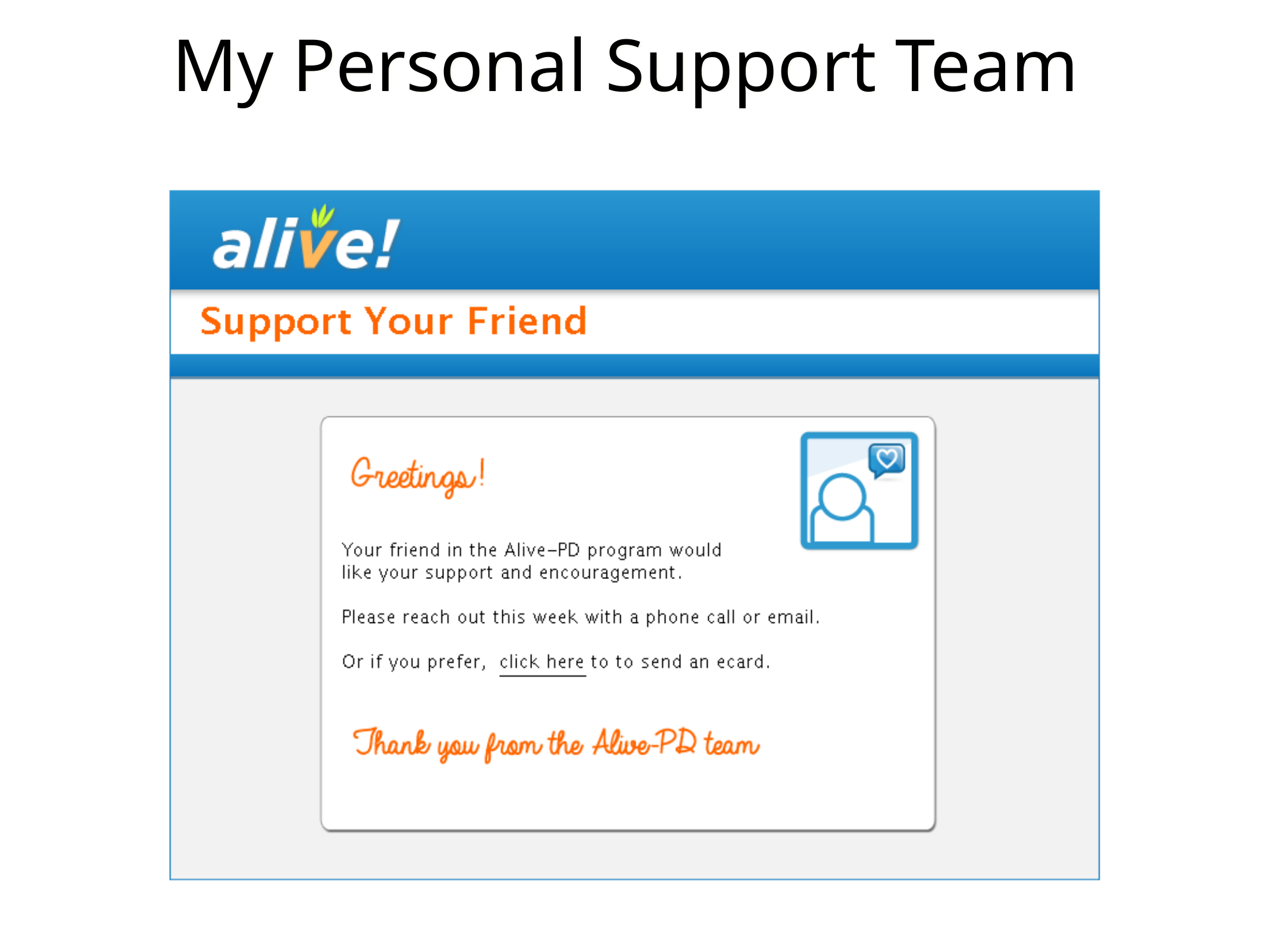

# My Personal Support Team

## Slide 38
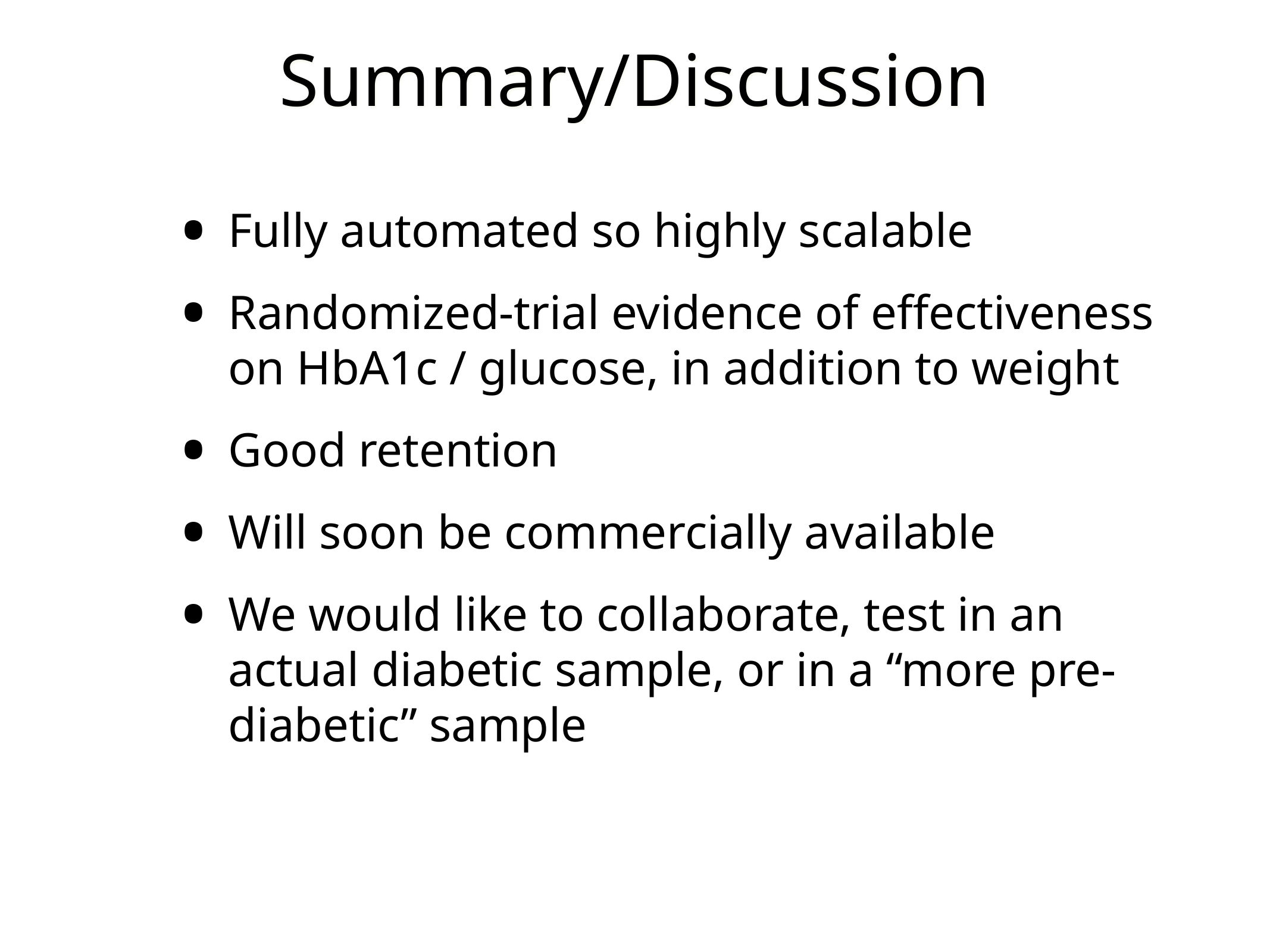

# Summary/Discussion
Fully automated so highly scalable
Randomized-trial evidence of effectiveness on HbA1c / glucose, in addition to weight
Good retention
Will soon be commercially available
We would like to collaborate, test in an actual diabetic sample, or in a “more pre-diabetic” sample

## Slide 39
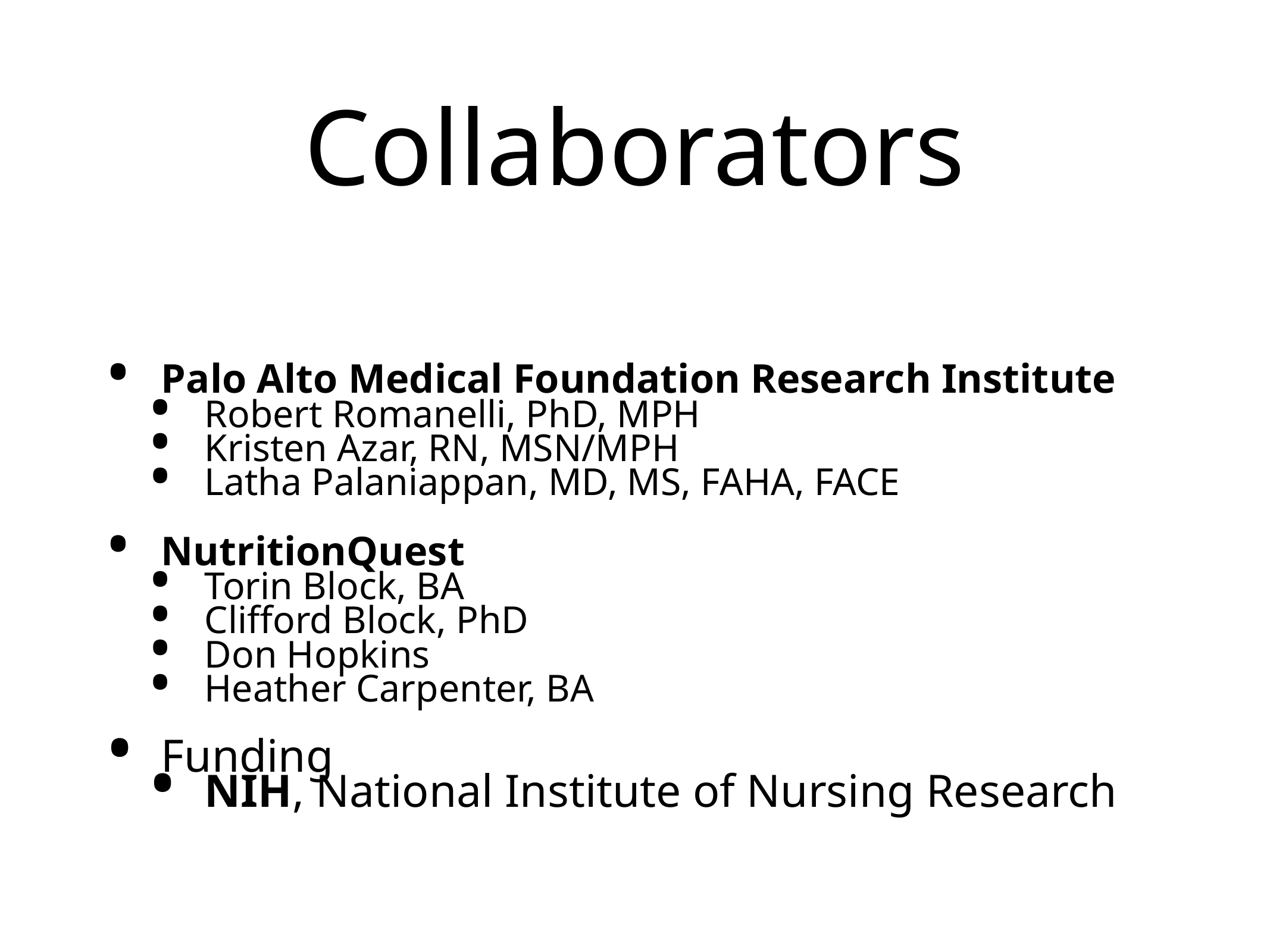

# Collaborators
Palo Alto Medical Foundation Research Institute
Robert Romanelli, PhD, MPH
Kristen Azar, RN, MSN/MPH
Latha Palaniappan, MD, MS, FAHA, FACE
NutritionQuest
Torin Block, BA
Clifford Block, PhD
Don Hopkins
Heather Carpenter, BA
Funding
NIH, National Institute of Nursing Research

## Slide 40
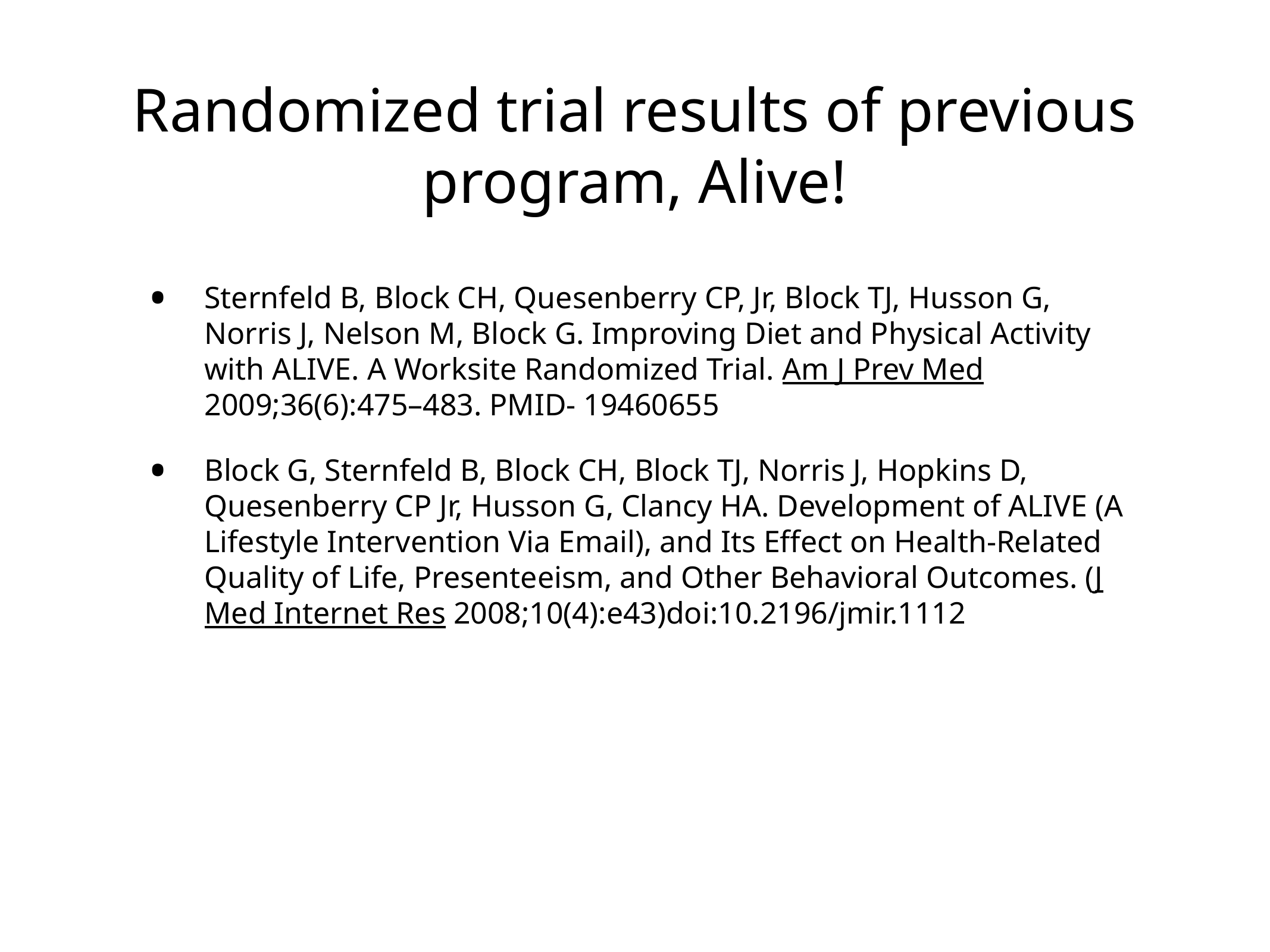

# Randomized trial results of previous program, Alive!
Sternfeld B, Block CH, Quesenberry CP, Jr, Block TJ, Husson G, Norris J, Nelson M, Block G. Improving Diet and Physical Activity with ALIVE. A Worksite Randomized Trial. Am J Prev Med 2009;36(6):475–483. PMID- 19460655
Block G, Sternfeld B, Block CH, Block TJ, Norris J, Hopkins D, Quesenberry CP Jr, Husson G, Clancy HA. Development of ALIVE (A Lifestyle Intervention Via Email), and Its Effect on Health-Related Quality of Life, Presenteeism, and Other Behavioral Outcomes. (J Med Internet Res 2008;10(4):e43)doi:10.2196/jmir.1112
